# In Vitro Characterization of a Tissue Renin-Angiotensin System in Human Nucleus Pulposus Cells

**DOI:** 10.3390/cells11213418

**Published:** 2022-10-28

**Authors:** Babak Saravi, Zhen Li, Valentina Basoli, Sibylle Grad, Sonja Häckel, Christoph E. Albers, Mauro Alini, Hagen Schmal, Peter Obid, Gernot Lang

**Affiliations:** 1 Department of Orthopedics and Trauma Surgery, Medical Centre—Albert-Ludwigs-University of Freiburg, Faculty of Medicine, Albert-Ludwigs-University of Freiburg, Hugstetterstrasse 55, 79106 Freiburg, Germany; 2 AO Research Institute Davos, 7270 Davos, Switzerland; 3 Department of Orthopaedic Surgery and Traumatology, Inselspital, Bern University Hospital, University of Bern, Freiburgstrasse 18, 3010 Bern, Switzerland

**Keywords:** intervertebral disc, inflammation, degeneration, renin-angiotensin system, therapy, angiotensin-converting enzyme 2

## Abstract

Low back pain is a clinically highly relevant musculoskeletal burden and is associated with inflammatory as well as degenerative processes of the intervertebral disc. However, the pathophysiology and cellular pathways contributing to this devastating condition are still poorly understood. Based on previous evidence, we hypothesize that tissue renin-angiotensin system (tRAS) components, including the SARS-CoV-2 entry receptor angiotensin-converting enzyme 2 (ACE2), are present in human nucleus pulposus (NP) cells and associated with inflammatory and degenerative processes. Experiments were performed with NP cells from four human donors. The existence of angiotensin II, angiotensin II type 1 receptor (AGTR1), AGTR2, MAS-receptor (MasR), and ACE2 in human NP cells was validated with immunofluorescent staining and gene expression analysis. Hereafter, the cell viability was assessed after adding agonists and antagonists of the target receptors as well as angiotensin II in different concentrations for up to 48 h of exposure. A TNF-α-induced inflammatory in vitro model was employed to assess the impact of angiotensin II addition and the stimulation or inhibition of the tRAS receptors on inflammation, tissue remodeling, expression of tRAS markers, and the release of nitric oxide (NO) into the medium. Furthermore, protein levels of IL-6, IL-8, IL-10, and intracellular as well as secreted angiotensin II were assessed after exposing the cells to the substances, and inducible nitric oxide synthase (iNOS) levels were evaluated by utilizing Western blot. The existence of tRAS receptors and angiotensin II were validated in human NP cells. The addition of angiotensin II only showed a mild impact on gene expression markers. However, there was a significant increase in NO secreted by the cells. The gene expression ratios of pro-inflammatory/anti-inflammatory cytokines IL-6/IL-10, IL-8/IL-10, and TNF-α/IL-10 were positively correlated with the AGTR1/AGTR2 and AGTR1/MAS1 ratios, respectively. The stimulation of the AGTR2 MAS-receptor and the inhibition of the AGTR1 receptor revealed beneficial effects on the gene expression of inflammatory and tissue remodeling markers. This finding was also present at the protein level. The current data showed that tRAS components are expressed in human NP cells and are associated with inflammatory and degenerative processes. Further characterization of the associated pathways is warranted. The findings indicate that tRAS modulation might be a novel therapeutic approach to intervertebral disc disease.

## 1. Introduction

Low back pain (LBP) is a musculoskeletal condition associated with pain and discomfort below the costal margins and above the inferior gluteal fold [1]. LBP is one of the most frequent reasons patients seek advice in the physician’s office. LBP prevalence increased from 377.5 million in 1990 to 577.9 million in 2017 [2]. As it is the leading cause of absenteeism from work, these epidemiological data translate to a substantial socioeconomic burden for healthcare systems globally [2]. Although the pathophysiology of low back pain is not fully understood, the current evidence suggests that an inflammatory and degenerative process within the intervertebral discs (intervertebral disc degeneration, IDD) is a major contributor to LBP [3]. The intervertebral disc (IVD) consists of fibrocartilaginous tissue with the central gelatinous nucleus pulposus (NP) and the surrounding anulus fibrosus (AF) [4]. The IVD is avascular in its native state and primarily innervated at the outer third of the AF [5,6]. Nevertheless, as soon as inflammatory and degenerative processes are present, vascularization and innervation of the whole IVD, including the NP, can be found [5,7]. The pathophysiological theory of this vascular and nerve ingrowth and the subsequent progression of IDD are complex, involving several pathways and cell types. Convincing evidence indicates that the interplay between initial inflammatory stimulus, vascular and neural ingrowth, and the subsequent invasion of immune cells mediating inflammation through interaction with the resident IVD cells, are building the main etiological pillars of progressive IDD and, subsequently, low back pain [3,5,8]. However, because the vascular ingrowth within the framework of IDD leads to access of NP cells to circulating molecules and immune cells, according to the current theory of the downfall of the immune privilege in IDD [9], the progression of IDD might be associated with unknown relevant, interactive pathways between the circulatory system and NP cells.

The renin-angiotensin system (RAS) is an important systemic regulator of salt and water balance as well as a modulator of systemic vascular resistance [10]. In recent years, studies have found local and intracellular components of this system in several human tissues [11]. Thus, the term “tissue renin-angiotensin system” (tRAS) was introduced to distinguish the local and intracellular pathways from the classical systemic role of the RAS [11,12]. The main receptors of this system are the angiotensin II type 1 receptor (AGTR1) as well as its counterparts, the angiotensin II type 2 receptor (AGTR2) and the Mas-receptor (MasR) [13]. The main effector is angiotensin II (AngII), which is generated through the angiotensin-converting enzyme (ACE) from angiotensin I (AngI) and can bind to both the AGTR1 and AGTR2 receptors. AngI itself can be generated from angiotensinogen (AGT) through renin or renin-like enzymes, such as cathepsin D (CTSD). The angiotensin-converting-enzyme 2 (ACE2), which recently became a focus due to its role as the SARS-CoV-2 virus entry receptor, can reduce local AngII concentrations by converting AngII to Ang1–7, which then functions as an agonist on the MasR [14]. The ACE/AngII/AGTR1 axis is currently considered the pathological axis, whereas the ACE2/AGTR2/MasR axis is considered the protective axis of the renin-angiotensin system [11,13]. Interestingly, numerous publications indicate musculoskeletal problems and low back pain in patients affected by COVID-19, although evidence to date is lacking regarding the existence of ACE2 in IVDs [15,16,17,18]. A downregulation of ACE2 could also affect the expression of all other components of tRAS and result in dysregulation of RAS related to the severity of COVID-19 [19,20]. The balance is disturbed after infection with SARS-CoV-2, but the mechanism by which tRAS dysregulation occurs is still unclear, and different scenarios have been proposed. As AngII levels increase, the classical AngII/AT1R signaling cascade is activated [11,21,22]. As a result, a strong pro-inflammatory and fibrotic response is triggered, which is likely to cause acute lung injury [14]. The downregulation of ACE2 results in the loss of Ang-(1–7) and the inhibition of downstream signaling pathways that have a protective role in the non-classical RAS axis [12]. This reveals an overactivation of the pathological axis and a deactivation of the protective axis. It is supported by a series of papers [23,24,25,26,27]. More in-depth information regarding the link between tRAS and COVID-19 can be found in recent reviews for interested readers [11,23,28].

In 2013, Morimoto et al. found components of the tRAS (ACE, AngII, AGTR1, AGTR2, CTSD) in mouse IVDs [29]. In 2020, Li et al. first described the expression of angiotensin II in human IVD tissues, found relevant gene expressions of AGTR1, AGTR2, AGT, and CTSD, and reported that the expression of these markers was associated with IDD [30]. In our previous study, an inhibitor of AGTR1 (Losartan) revealed anti-inflammatory and anti-degenerative effects on human NP cells [31]. Modulation of tRAS can be performed in several ways. One therapeutic option would be to inhibit the pathological tRAS axis with AGTR1 inhibitors (e.g., losartan, candesartan). Another way would be to stimulate the protective axis with agonists of AGTR2 (e.g., compound 21/C21) or MAS1 (e.g., Ang1–8). Furthermore, ACE2 addition (e.g., rhACE2), ACE2 activators (e.g., xanthenon, DIZE), ACE-inhibitors (e.g., captopril) are counted as tRAS modulation drugs [32]. When considering the listed evidence from the literature, we hypothesize that the components of the tRAS are expressed in human NP cells and are associated with inflammatory and degenerative processes. In this study, we aim to validate the expression of all major tRAS components in human NP cells on both protein and gene expression levels and characterize these components functionally by modulating their activity. The overall aim of this study is to investigate whether the modulation of tRAS might be of relevance for treating IDD.

## 2. Materials and Methods

### 2.1. Human NP Cells Isolation and Expansion

The Swiss Human Research Act does not apply to research that utilizes anonymized biological material and/or anonymously collected or anonymized health-related data. Therefore, this project did not need to be approved by an ethics committee. Patients’ general consent was obtained, which also covers the anonymization of health-related data and biological material.

Human NP cells were derived from four human donors who underwent spinal surgery at InselSpital, Bern, Switzerland. The discs were obtained from male donors, 50–70 years old, and Pfirrmann grade III–IV. NP tissue was incubated with red blood cell lysis buffer (155 mM NH_4_Cl, 10 M KHCO_3_, and 0.1 mM EDTA in Milli-Q water) for 5 min on a shaker at room temperature to remove contamination with blood cells. Hereafter, the tissue was washed with phosphate-buffered saline (PBS), and the chopped tissue was digested with 0.2% *w*/*v* pronase (Roche, Basel, Switzerland) in alpha minimum essential medium (αMEM, Gibco, Life Technologies, Carlsbad, CA, USA) for 1 h, followed by 65 U/mL collagenase type II (Worthington, Columbus, OH, USA) in αMEM/10% fetal calf serum (FCS, Corning, Corning, NY, USA) at 37 °C for 12–14 h. The tissue debris was then filtered through a 100-μm cell strainer to obtain a single-cell suspension. Cells (10,000 cells/cm^2^) were seeded with αMEM supplemented with 10% FCS, 100 U/mL penicillin and 100 µg/mL streptomycin (1% P/S, Gibco). A hypoxic condition of 2% O_2_ was maintained during cell culture. The culture medium was changed twice a week. Cells were detached by using a dissociation buffer composed of 0.05% trypsin/EDTA (Gibco) for 5 min at 37 °C when they reached approximately 80% confluency. Cells were subcultured at a cell density of 3000 cells/cm^2^ for expansion. Passage 1 NP cells were cryopreserved in liquid N2 for subsequent experiments. After thawing, NP cells were expanded with high-glucose Dulbecco’s minimum essential medium (high glucose DMEM, Sigma Aldrich, St. Louis, MO, USA) and 10% FCS. Passage 3 NP cells were used for the experiments.

### 2.2. Experimental Design

Experiments were performed to evaluate the impact of angiotensin II, AGTR1 inhibition, AGTR2 stimulation, and MasR stimulation on inflammation, tissue remodeling, and expression of tRAS markers in human NP cells. Angiotensin II (Sigma Aldrich, St. Louis, MO, USA, A9525-5MG), the AGTR1 antagonist candesartan (Sigma, SML0245-10MG), the MasR agonist AVE 0991 (Lucerna-Chem, Luzern, Switzerland, HY-15778-5MG), and the AGTR2 agonist CGP-42112A (Sigma Aldrich, C160-1MG) were supplemented at different concentrations in the culture medium. Four donors with three replicates per donor, group, and concentration were assessed in this experiment. After detaching cells at 80% confluency, passage 3 NP cells were seeded in 12-well plates at a density of 20,000 cells/well. Cells were seeded in 2 mL DMEM with 10% FCS to allow cell attachment. On the day of cell seeding (Day 0, baseline), samples were taken for normalization. After 24 h, the medium was changed to the experimental medium. The control group was cultured with 2 mL DMEM, 1% non-essential amino acids, 50 µg/mL L-ascorbic acid 2-phosphate, and 1% ITS+. Angiotensin II (0.1 µM, 10 µM), candesartan (1 µM, 10 µM, 100 µM), AVE 0991 (1 µM, 10 µM, 100 µM), or CGP-42112A (0.1 µM, 1 µM, 10 µM) were added to the respective treatment groups at the mentioned concentrations. Human recombinant tumor necrosis factor alpha (TNF-α) (10 ng/mL; R&D Systems Europe, Ltd., Abingdon, UK, 210-TA-020) was added simultaneously to induce a pro-inflammatory stimulus in human NP cells. The TNF-α + DMSO (Sigma, D2438) group was included as the solvent control. The study group design is listed in Table 1.

Cells from four donors were used for the experiments, and all groups had three experimental replicates. Two concentrations of angiotensin II and three concentrations of CGP42112A, AVE 0991, and candesartan were investigated. The range of concentrations chosen was based on investigations in other cell types and in vitro experiments reported in the literature, as no evidence was available for the use of these drugs in NP cells [13,33,34,35,36]. After treatment for 24 h and 48 h of exposure, the medium was collected to analyze the nitric oxide (NO) content by utilizing the Griess reagent system according to the manufacturer’s instruction (Ref.: G2930, Promega, Madison, WI, USA). Furthermore, the collected medium was used to assess inflammatory marker concentrations with ELISA (see below). Cells were then lysed in TRI reagent (Molecular Research Center, Cincinnati, OH, USA) and PolyAcrylCarrier (Molecular Research Center, Cincinnati, OH, USA) and stored at −80 °C for gene expression analysis.

### 2.3. Cell Metabolic Activity

A cell metabolic activity analysis was performed to investigate the potential cytotoxic effects of angiotensin II, the AGTR1 antagonist candesartan, the MasR agonist AVE 0991, and the AGTR2 agonist CGP-42112A. This experiment used two donors with three technical replicates per donor, drug, and concentration. This experimental design was then triplicated to assess the effects after 24 h, 48 h, and 72 h of exposure. NP cells were seeded in 96-well plates at a density of 2000 cells per well with DMEM containing 1% ITS+, 1% non-essential amino acids (Sigma Aldrich), and 50 µg/mL L-ascorbic acid 2-phosphate (Sigma Aldrich) and incubated for 24 h. Hereafter, angiotensin II (0.1 µM, 1 µM, 10 µM), candesartan (1 µM, 10 µM, 100 µM), AVE 0991 (1 µM, 10 µM, 100 µM), or CGP-42112A (0.1 µM, 1 µM, 10 µM) were added to the respective treatment groups at the mentioned concentrations. Furthermore, 10 ng/mL TNF-alpha and the solvent used to dissolve the drugs (DMSO) (0.1 µL/mL, 1 µL/mL, 10 µL/mL) were added to the experimental groups according to the study design described in the previous section. After incubation for 24, 48, and 72 h, cells were washed with PBS and then exposed to the Cell Titer Blue^®^ reagent (Promega Corporation, Madison, WI, USA) diluted 1:5 in DMEM. Fluorescence intensity was determined with the Viktor 3 plate reader (Perkin Elmer, Waltham, MA, USA) after 4 h of incubation (ex/em 560/590 nm).

### 2.4. Immunofluorescent Staining

To validate the presence of angiotensin II, AGTR1, AGTR2, MasR, and ACE2, cells were detached at 80% confluency and seeded in black 96-well plates (µ-Plate 96-well black, Ibidi GmbH, Planneg, Germany). A feasibility study was first performed to determine the best concentrations of antibodies and reagents that could adequately visualize the target proteins. The final experiments were performed with four different donors in triplicate for each tRAS component. After 24 h, wells were washed twice with 200 µL PBS, and 400 µL blocking buffer (3 g BSA and 0.1 mL Triton X-100 in 100 mL PBS) was added to each well. After 20 min at room temperature, PBS was decanted, and 140 µL of primary antibody solution was added to the respective wells. Primary antibodies were diluted in a blocking buffer. The antibodies and dilution factors are shown in Table 2. The plates were then incubated for 1 h at 37 °C. Hereafter, wells were washed twice with PBS, and 140 µL secondary antibody (anti-rabbit, Alexa Fluor 488 green) solution at a dilution of 1:1000 was added to each well. PBS was used to dilute the secondary antibody. An additional well per donor and group was included as the negative control (secondary antibody only). Plates were again incubated for 1 h at 37 °C. Then, wells were washed twice in the dark, and 140 µL DAPI was added at a dilution of 1:1000. After 10 min at room temperature, cells were investigated by utilizing confocal microscopy.

### 2.5. Gene Expression Analysis

RNA extraction was performed with TRI reagent, and RNA content was quantified by using a NanoDrop ND-1000 spectrophotometer (Thermo Fisher Scientific Inc., Waltham, MA, USA). SuperScript VILO cDNA Synthesis Kit (Life Technologies, Carlsbad, CA, USA) was then used for reverse transcription, followed by real-time quantitative PCR (q-PCR) by using the TaqMan™ reagent with a 10-µL reaction volume. The gene expression level of matrix metalloproteinase-1 and -3 (MMP-1, -3), interleukin 6, 8, and 10 (IL-6, -8, -10), TNF-α, toll-like receptor 4 (TLR4), interleukin-1-beta (IL1-b), collagen 1 (COL1A1) and 2 (COL2A1), and ACAN were measured as markers for inflammation and tissue remodeling. The gene expression analysis of tRAS markers included: angiotensinogen (AGT), angiotensin-converting enzyme (ACE), angiotensin-converting enzyme 2 (ACE2), renin-like tRAS equivalent cathepsin D (CTSD), the angiotensin II receptor type I (AGTR1) and type 2 receptor (AGTR2), and the Mas-receptor (MAS1). RPLP0 was used as a housekeeping gene in each sample (Table 3). The 2^(−DeltaDeltaCt) method was applied for relative quantification with RPLP0 as the endogenous control, and the results are shown as fold change compared to the control group.

### 2.6. Enzyme-Linked Immunosorbent Assay (ELISA)

Angiotensin II (Angiotensin II EIA Kit, Sigma Aldrich, Catalog #RAB0010-1KT), IL-6 (ELISA DuoSet^®^, R&D Systems, Catalog #DY206-05), IL-8 (ELISA DuoSet^®^, R&D Systems, Catalog #DY208-05), and IL-10 (ELISA DuoSet^®^, R&D Systems, Catalog #DY217B-05) amounts secreted in the medium by the cells after 24 h of exposure were measured with enzyme-linked immunosorbent (ELISA) kits. The assays were done according to the manufacturer’s instructions. For IL-6, IL-8, and IL-10, capture antibodies were diluted to the working concentration provided in the manufacturer’s instruction, and the microplates were coated with the diluted capture antibody, sealed, and incubated at room temperature overnight. For angiotensin II, the delivered plates had already been coated with a capture antibody. Hereafter, each well was aspirated and washed with a wash buffer. The plates were then blocked by adding a blocking buffer to each well. The plate was incubated at room temperature for a minimum of 1 h. The wash step was repeated, and samples or standards were diluted in reagent diluent. Then, the plate was sealed and incubated for 2 h at room temperature. The wash step was repeated, and a working dilution of streptavidin-HRP was added to each well. The well was sealed and incubated at room temperature for 20 min and protected from light. The wash step was repeated, and 100 µL of substrate solution (1:1 mixture of H_2_O_2_ and tetramethylbenzidine) was added to each well. The plate was incubated for 20 min at room temperature and protected from light. Finally, the stop solution (2N H_2_SO_4_) was added to each well, and the optical density of each well was determined by using a microplate reader at 450 nm with wavelength correction at 570 nm. A separate experiment was conducted to evaluate the intracellular angiotensin II concentrations in the experimental groups. A protease inhibitor cocktail (Sigma Aldrich, P8340-5ML) was added in a dilution of 1:100 to RIPA buffer (Sigma Aldrich) to prepare a cell lysis solution. After aspirating the medium from the well plates and washing the wells by using PBS, 120 µL of the cell lysis solution was added to each well. After thorough mixing by pipetting up and down, lysed cells were collected into protein low-binding tubes and analyzed with ELISA as described above.

### 2.7. Western Blot

Western blot was performed to quantify the translation of inducible nitric oxide synthase (iNOS) in the different experimental groups. Cells were seeded in 100-mm petri dishes (one million cells per dish), and DMEM + 10% FCS was added to attach cells. After 24 h, the medium was changed to DMEM, 1% non-essential amino acids, 50 µg/mL L-ascorbic acid 2-phosphate, and 1% ITS+ and cells were exposed to the respective drug group or medium only (control). After 24 h of exposure, cells were detached by using the detaching solution and cell scraper and collected into centrifugation tubes. Hereafter, cells were centrifuged at 400× *g* for 5 min, and after aspiration of the supernatant, the pellet was washed with 1 mL PBS, transferred to 1.5 mL Eppendorf tubes, and centrifuged again at 400× *g* for 5 min. The supernatant was decanted, and 100 µL of RIPA buffer + 1% protease inhibitor (Sigma Aldrich) was added to each tube. Protein content was quantified by utilizing bicinchoninic acid assay (BCA) (Thermo-Fisher, #23225) according to the manufacturer’s instructions. Proteins were then loaded by using Laemmli buffer. Hereafter, samples were run by using precast TGX Mini-Protean SDS-PAGE gels (BioRad, with 4–15% acrylamide/bisacrylamide) at 120 V for 45 min in TGX buffer. Gels after the run were transferred to a 0.2 µm nitrocellulose membrane by using trans-blot turbo (Biorad, #1704159), blocked with 6% BSA in TBST containing 0.1% Tween 20, incubated with iNOS primary antibody (R&D systems, MAB9502-SP) at a working concentration of 1 µg/mL, and then incubated with anti-secondary antibody (goat anti-mouse (Invitrogen, Waltham, MA, USA, #A28177), dilution 1:500). Magic XP Western standard (Invitrogen, #LC5602) and iBright standard (Invitrogen, LC5615) were used as reference standards. GAPDH was used as the control protein. ECL prime Western blotting detection reagent (cytiva, MA, USA, #RPN2232) was used as a detection reagent. ImageJ was used for Western blot quantification [37].

### 2.8. Statistical Analysis

Data distribution was assessed with the Shapiro–Wilk normality test. The differences were assessed by using two-way-ANOVA or non-parametric alternatives with subsequent multiple-comparison post hoc tests depending on the assumptions fulfilled by the data. Correlation analysis was performed by utilizing the Spearman rank correlation test and its coefficient Spearman’s rho. A two-sided *p*-value < 0.05 was considered significant. Statistical analyses were conducted in GraphPad Prism software, version 8.2.1 (GraphPad Software, Inc., San Diego, CA, USA).

## 3. Results

### 3.1. Evaluation of tRAS Protein Translation in Human NP Cells via Immunofluorescence

**Figure 1:**
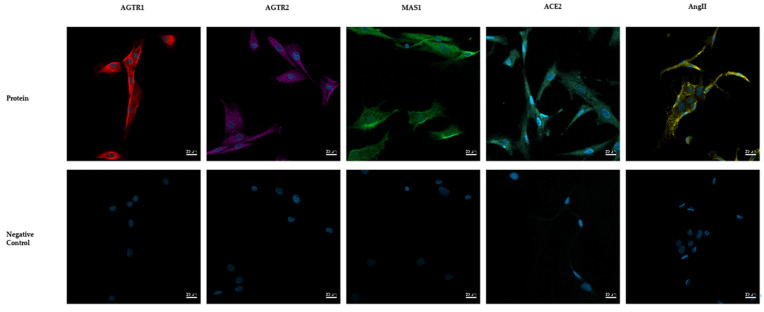
Immunofluorescence analysis of the angiotensin II type 1 (AGTR1) and 2 (AGTR2) and MAS1 receptor, as well as angiotensin II (AngII) and angiotensin-converting enzyme 2 (ACE2). Representative images are shown. *n* = 4 biological replicates with 2 technical replicates each were evaluated via immunofluorescence. DAPI was used to visualize cell nuclei. Scale bar, 20 μm.

Immunofluorescence was performed to validate AGTR1, AGTR2, and MAS1 receptors, as well as the main effector AngII and ACE2 in human NP cells. All mentioned receptors were expressed in human nucleus pulposus cells and showed a positive fluorescence signal (Figure 1).

### 3.2. Impact of Angiotensin II Addition on Human Nucleus Pulposus Cells

#### 3.2.1. Cell Metabolic Activity Analysis

**Figure 2:**
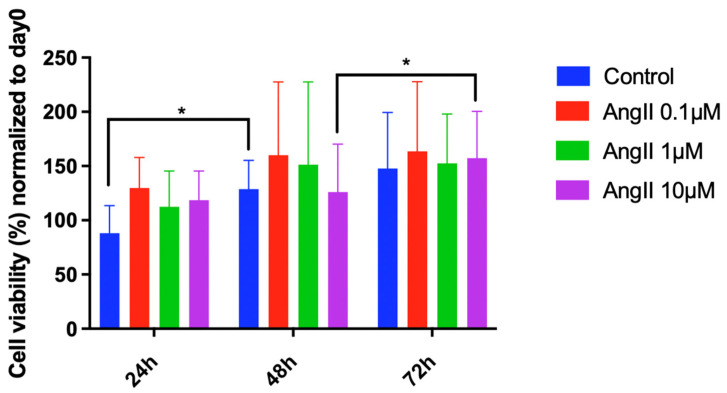
Cell metabolic activity analysis in human NP cells treated with angiotensin II (0.1 µM–10 µM) after 24 h, 48 h, and 72 h of exposure. Cell metabolic activity normalized to the baseline (day 0/beginning of drug exposure) is shown. *n* = 2 biological replicates and *n* = 3 technical replicates per donor were assessed. * *p* < 0.05.

Cell metabolic activity analysis was performed to evaluate the impact of different angiotensin II concentrations up to 72 h of exposure on human NP cell proliferation (Figure 2). Results revealed no cytotoxic effects of angiotensin II within the concentration and exposure range investigated.

#### 3.2.2. Gene Expression Analysis

Gene expression analysis was performed to evaluate whether angiotensin II addition in two different concentrations (0.1 µM (“ATlow”) and 10 µM (“AThigh”) caused significant effects on a range of gene expression markers in human NP cells after 24 h (Supplementary Figure S1) and 48 h (Supplementary Figure S2) of exposure. Furthermore, we assessed whether inflammatory conditions (TNF-α + AThigh and TNF-α + ATlow, respectively) would lead to a different reaction of NP cells to angiotensin II addition. The results are shown in Supplementary Figures S1 (24 h) and S2 (48 h). The tissue degenerative markers matrix-metalloproteinase I (MMP1) and III (MMP3), as well as the inflammatory markers (IL-6, IL-8, TNF-α, TLR4) and anti-inflammatory markers (IL-10), significantly increased by TNF-α addition at both time points. Interestingly, tRAS markers (AGTR1, AGTR2, ACE, and MAS1) were significantly increased by TNF-α at both time points. Aggrecan (ACAN) expression was not affected in our in vitro model. COL1A1 expression was significantly increased in the TNF-α + ATlow and TNF-α + AThigh group after 24 h but not 48 h of exposure. We further observed a significant increase in IL-8 expression for the TNF-α + AThigh group compared to the TNF-α-only group. Other gene expression markers were not significantly affected by angiotensin II addition.

**Figure 3:**
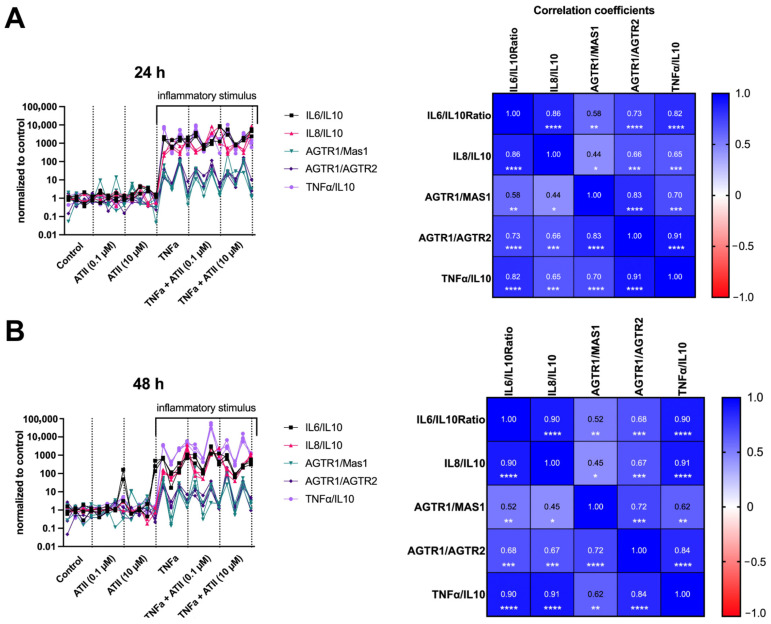
Illustration of tRAS axis ratios (AGTR1/AGTR2, AGTR1/MAS1) [11] and inflammatory to anti-inflammatory gene expression markers (Il-6/IL-10, IL-8/IL-10, and TNF-α/IL-10) after 24 h (**A**) and 48 h (**B**) of drug or control exposure. Ratios of pro-inflammatory to anti-inflammatory markers are indicators for the inflammatory state of the intervertebral disc and low back pain [38,39]. AGTR1/AGTR2 and AGTR1/MAS1 gene expression ratios were positively correlated with the pro-inflammatory/anti-inflammatory marker ratios Il-6/IL-10, IL-8/IL-10, and TNF-α/IL-10. Spearman correlation coefficients are shown in the matrix. Fold change values obtained from the 2^(−DeltaDeltaCt) method and normalized to the baseline (day 0) are shown. *n* = 4 biological replicates and *n* = 3 technical replicates per donor were assessed. Inflammatory stimulus indicates the groups exposed to TNF-α. Correlation of ratios was assessed by taking the mean from the technical replicates for each group of the respective donors and calculating the correlation coefficient between the ratios (*n* = 24 per ratio; 4 donors x 6 groups (with the mean used for the three technical replicates per group). Lines are connected for better visual differentiation between the ratios. Each symbol represents one replicate per group and donor. * *p* < 0.05; ** *p* < 0.01; *** *p* < 0.001; **** *p* < 0.0001.

We further evaluated whether there was a correlation between the pathological to protective tRAS axis gene expressions (AGTR1/AGTR2, AGTR1/MAS1) and inflammatory to anti-inflammatory gene expression markers (Il-6/IL-10, 1L-8/IL-10, and TNF-α/IL-10) in this experimental setting. Ratios of pro-inflammatory to anti-inflammatory markers (IL-6/IL-10, IL-8/IL-10, and TNF-α/IL-10) are indicators of the inflammatory state of the IVD and low back pain [38,39]. Our results revealed that AGTR1/AGTR2 and AGTR1/MAS1 gene expression ratios were significantly correlated with the pro-inflammatory/anti-inflammatory marker ratios Il-6/IL-10, IL-8/IL-10, and TNF-α/IL-10 (Figure 3). After the inflammatory stimulus (TNF-α groups), both the ratio of the pathological/protective axis and inflammatory/anti-inflammatory marker ratios increased significantly. Angiotensin II did not significantly enhance this inflammatory state for the ratios examined. However, these alterations in the gene expression ratios were positively and highly correlated, as seen in Figure 3. As expected, there was a significant positive correlation between the Il-6/IL-10, IL-8/IL-10, and TNF-a/IL-10 ratios. These correlations were higher for the 48 h of exposure investigations (Figure 3). In contrast, there were higher correlations between the pathological/protective tRAS axis gene expressions and the inflammatory/anti-inflammatory marker ratios after 24 h of exposure. The highest correlation with the pathological/protective tRAS axis gene expression was found for the TNF-α/IL-10 ratio (AGTR1/MAS1 and TNF-α/IL-10: Spearman’s rank correlation, rho = 0.70, *p* < 0.001; AGTR1/AGTR2 and TNF-α/IL-10: Spearman’s rank correlation, rho = 0.91, *p* < 0.0001).

#### 3.2.3. Nitrite Release from the Metabolism of Nitric Oxide

**Figure 4:**
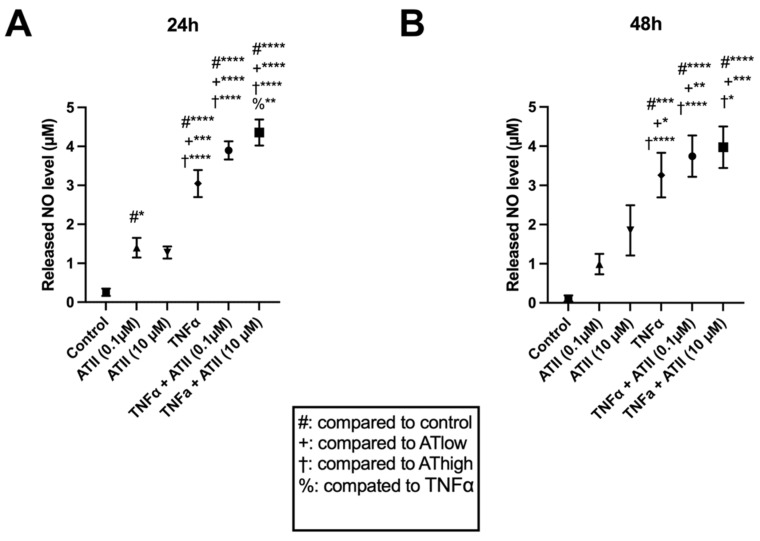
Illustration of indirect nitric oxide (NO_2_) concentrations in the medium for the study groups after 24 h (**A**) and 48 h (**B**) of drug/control exposure. Nitric oxide was measured indirectly from nitrite release from the metabolism of nitric oxide. *n* = 4 biological replicates and *n* = 3 technical replicates per donor were assessed. Mean and standard error of the mean (SEM) are shown. #,* *p* < 0.05; ** *p* < 0.01; *** *p* < 0.001; **** *p* < 0.0001.

We also evaluated the presence of nitrite metabolized from nitric oxide (NO_2_), an inflammation marker found in the medium after 24 h and 48 h of exposure, by Griess assay. TNF-α significantly increased the NO_2_ secretion at both time points. We also found a significant increase of NO_2_ secreted by the cells in the AngII exposed groups compared to the control (AngII versus control; *p* < 0.05) or TNF-α only group (TNF-α + AngII versus TNF-α; *p* < 0.01) after 24 h of exposure. However, this finding was absent after 48 h (Figure 4).

### 3.3. Impact of Pathological Axis (AGTR1) Inhibition versus Protective Axis (AGTR2, MAS1) Stimulation on Human Nucleus Pulposus Cells

#### 3.3.1. Cell Metabolic Activity Analysis

**Figure 5:**
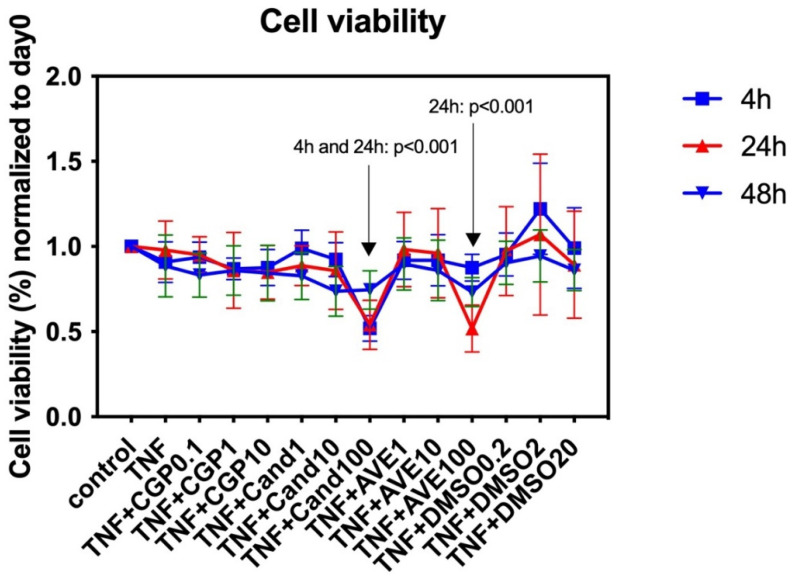
Cell metabolic activity analysis in human NP cells for candesartan (“Cand”) (1 µM, 10 µM, 100 µM), the MAS-receptor agonist AVE 0991 (“AVE”) (1 µM, 10 µM, 100 µM), and the AGTR2-receptor agonist CGP-42112A (“CGP”) (0.1 µM, 1 µM, 10 µM) with 4 h, 24 h, and 48 h of exposure. DMSO (0.1 µL/mL, 1 µL/mL, 10 µL/mL; shown in µL volume on the *x*-axis) was used as the solvent control. Cell viability normalized to the baseline (day 0/timepoint of drug exposure) is shown. *n* = 2 biological replicates and *n* = 3 technical replicates per donor were assessed. Mean and standard deviation (SD) are shown.

Cell metabolic activity analysis was performed to evaluate the impact of the AGTR1 inhibitor candesartan (1 µM, 10 µM, 100 µM), the MAS-receptor agonist AVE 0991 (1 µM, 10 µM, 100 µM), and the AGTR2-receptor agonist CGP-42112A (0.1 µM, 1 µM, 10 µM) with up to 48 h of exposure on human NP cells’ proliferation (Figure 5). Concentration ranges and exposure times were chosen based on a preliminary literature search for other cell types, as no evidence was available for human NP cells. We found significant cytotoxic effects for the 100-µM candesartan group (4 h and 24 h of exposure, but not 48 h of exposure) and 100 µM AVE 0991 group (24 h of exposure). CGP-42112A (“CGP”) (0.1 µM, 1 µM, 10 µM) did not reveal apparent cell proliferative or cytotoxic effects within the concentration and exposure range investigated.

#### 3.3.2. Gene Expression Analysis

Gene expression analysis was performed to evaluate whether the proposed protective effects of the inhibition of the pathological axis (AGTR1 inhibition) and the stimulation of the protective axis (AGTR2 and MAS1 stimulation) are also found in human NP cells [11]. For this, we assessed the alterations of a range of gene expression markers in human NP cells after 4 h and 24 h of exposure in inflammatory settings (TNF-α addition). All gene expression analyses can be found in the Supplementary Figures S3 (4 h of exposure) and S4 (24 h of exposure).

##### Inflammation

**Figure 6:**
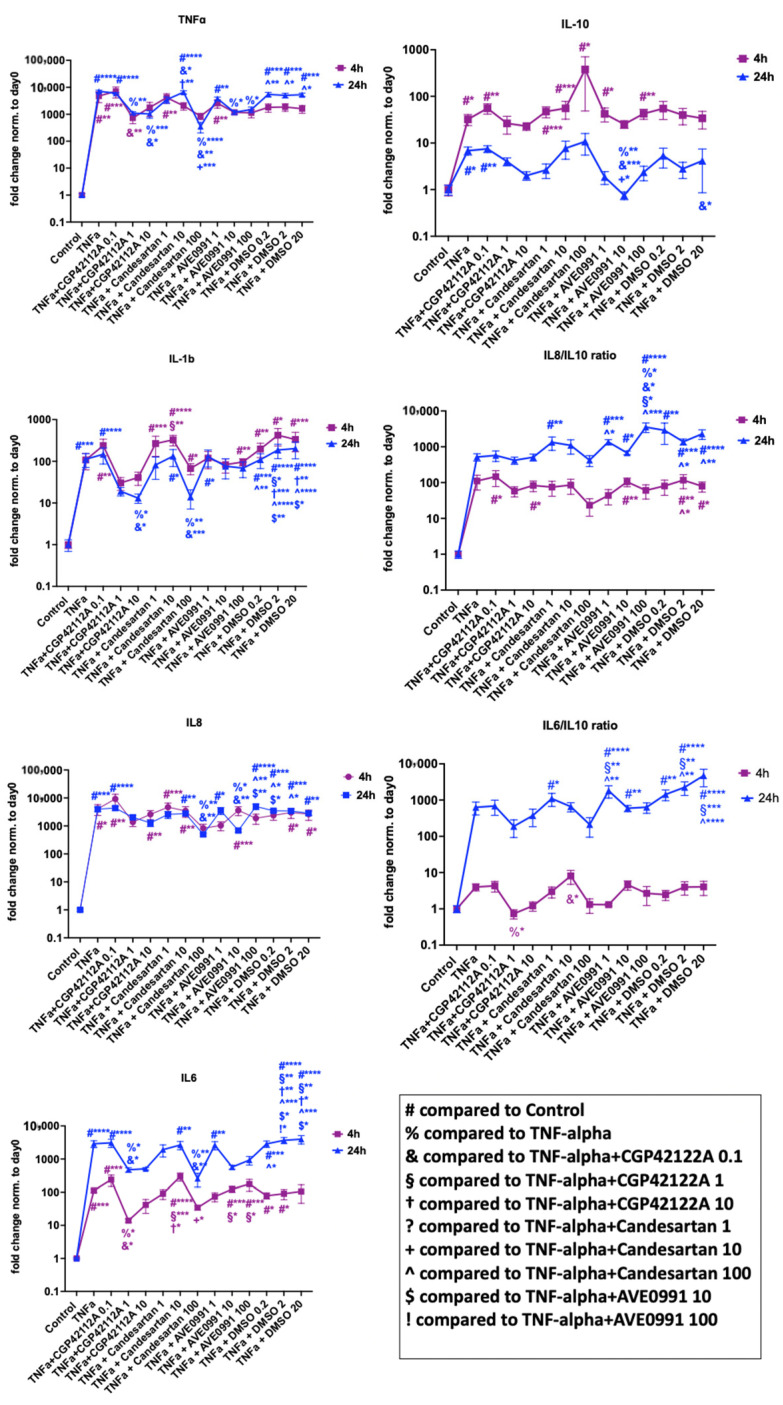
Gene expression analysis of inflammation-related markers in human NP cells for candesartan (1 µM, 10 µM, 100 µM), the MAS-receptor agonist AVE 0991 (1 µM, 10 µM, 100 µM), and the AGTR2-receptor agonist CGP-42112A (0.1 µM, 1 µM, 10 µM) with 4 h and 24 h of exposure in inflammatory settings (TNF-α addition). DMSO (0.1 µL/mL, 1 µL/mL, 10 µL/mL; shown in µL volume on the *x*-axis) was used as the solvent control. Fold change values normalized to the baseline (day 0) are shown. *n* = 4 biological replicates and *n* = 3 technical replicates per donor were assessed. Mean and standard error of the mean (SEM) are shown. * *p* < 0.05; ** *p* < 0.01; *** *p* < 0.001; **** *p* < 0.0001.

The gene expression analysis results of inflammation-related markers are shown in Figure 6. TNF-α was able to induce both the expression of inflammatory (IL-1β, IL-6, IL-8, TNF-α) and anti-inflammatory (IL-10) markers. The AGTR2 receptor agonist CGP-42112A (1 µM and/or 10 µM but not 0.1 µM) was able to reverse TNF-α-induced gene expression significantly, namely the IL-6, IL6/IL10 ratio (1 µM, 4 h exposure: *p* < 0.05), and IL-1β, whereas IL-10 and the IL-8/IL-10 ratio was not significantly affected. Furthermore, we observed a significant anti-inflammatory effect for the AGTR1 inhibitor candesartan 100-µM group after 24 h of exposure. Finally, the MAS1 receptor agonist AVE 0991 in 10 µM and/or 100 (but not 1) µM significantly reversed the TNF-α-induced gene expression of TNF-α (10 µM and 100 µM; *p* < 0.05) and IL-8 (10 µM; *p* < 0.05) after 24 h of exposure. However, we also found a significant downregulation of the anti-inflammatory marker IL-10 for AVE 0991 in 10 µM concentration and an upregulation of the IL-8/IL-10 ratio for AVE 0991 in 100 µM concentration after 24 h of exposure.

##### Tissue Degeneration and Phenotype Modulation

**Figure 7:**
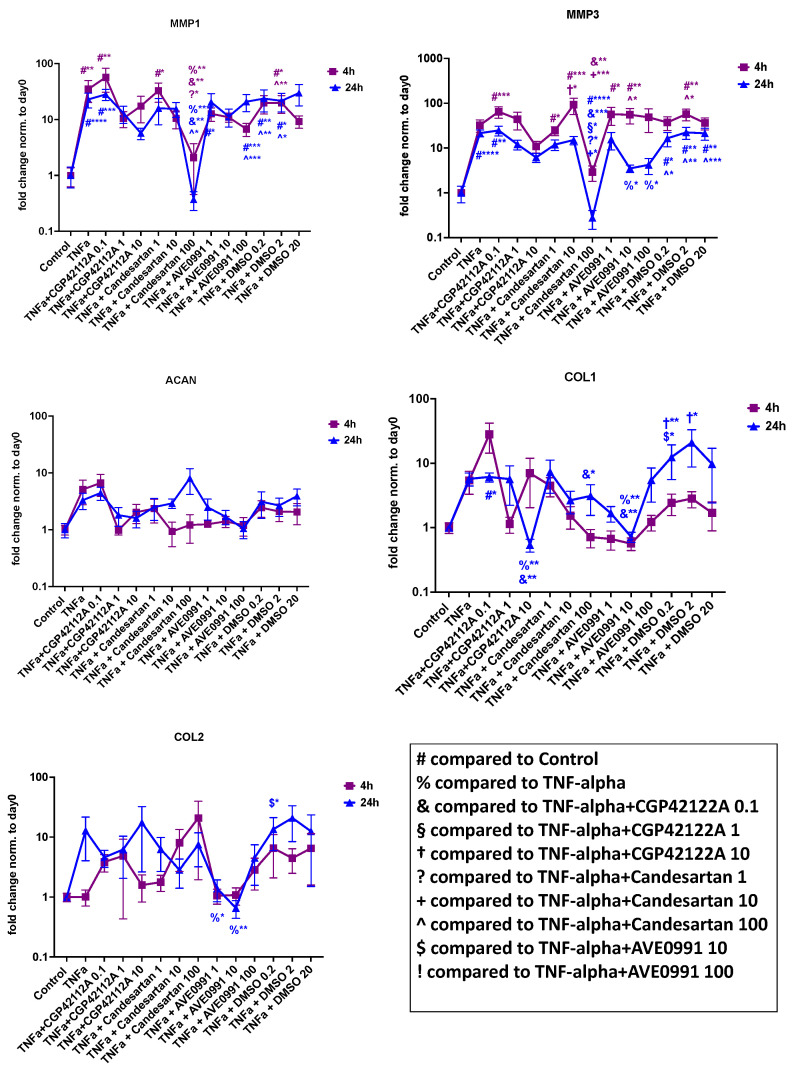
Gene expression analysis of tissue-degenerative and phenotype-modulating markers in human NP cells for candesartan (1 µM, 10 µM, 100 µM), the MAS-receptor agonist AVE 0991 (1 µM, 10 µM, 100 µM), and the AGTR2-receptor agonist CGP-42112A (0.1 µM, 1 µM, 10 µM) with 4 h and 24 h of exposure in inflammatory settings (TNF-α addition). DMSO (0.1 µL/mL, 1 µL/mL, 10 µL/mL; shown in µL volume on the *x*-axis) was used as the solvent control. Fold change values normalized to the baseline (day 0) are shown. *n* = 4 biological replicates and *n* = 3 technical replicates per donor were assessed. Mean and standard error of the mean (SEM) are shown. * *p* < 0.05; ** *p* < 0.01; *** *p* < 0.001; **** *p* < 0.0001.

The gene expression analysis results of tissue-degenerative and phenotype markers are depicted in Figure 7. TNF-α was able to significantly increase the expression of MMP1 and MMP3. The findings also indicated that TNF-α might increase the expression of phenotype markers (COL1A1, COL2A1, ACAN); however, this finding missed statistical significance at both time points. AGTR2 receptor agonist CGP-42112A (10 µM but not 0.1 and 1 µM) was able to significantly reverse TNF-α induced gene expression of COL1A1, whereas COL2A1 and ACAN were not significantly affected after 24 h of exposure. MAS1 receptor agonist AVE 0991 in 10 µM (but not 1 µM and 100 µM) reversed the TNF-α-induced upregulation of COL1A1 after 24 h of exposure. In addition, AVE 0991 at 1 µM and 10 µM (but not 100 µM) reversed the TNF-α-induced upregulation of COL2A1. Furthermore, we observed a significant anti-degenerative effect (MMP1 and MMP3 downregulation) for the AGTR1 inhibitor candesartan 100-µM group after 24 h of exposure. This was also seen for the MAS1 receptor agonist AVE 0991 at 10 µM and 100 µM concentrations (but not 1 µM) after 24 h of exposure.

##### tRAS Markers

**Figure 8:**
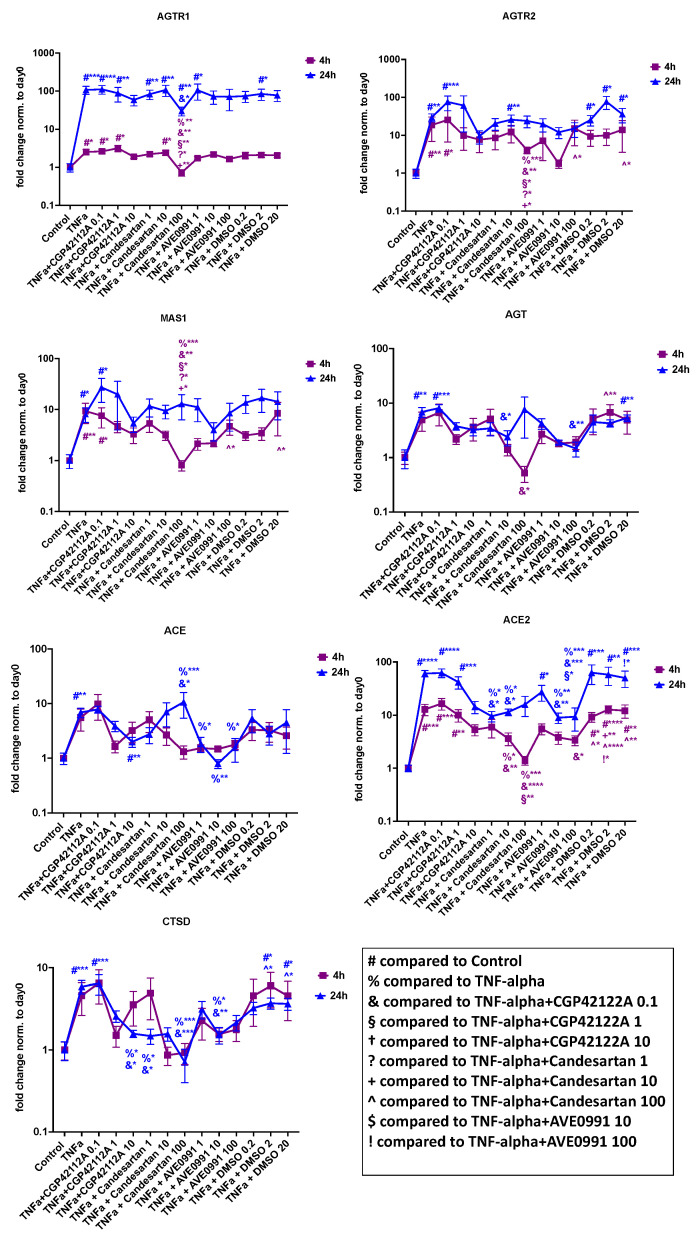
Gene expression analysis of tRAS markers in human NP cells for candesartan (1 µM, 10 µM, 100 µM), the MAS-receptor agonist AVE 0991 (1 µM, 10 µM, 100 µM), and the AGTR2-receptor agonist CGP-42112A (0.1 µM, 1 µM, 10 µM) with 4 h and 24 h of exposure in inflammatory settings (TNF-α addition). DMSO (0.1 µL/mL, 1 µL/mL, 10 µL/mL; shown in µL volume on the *x*-axis) was used as the solvent control. Fold change values normalized to the baseline (day 0) are shown. *n* = 4 biological replicates and *n* = 3 technical replicates per donor were assessed. Mean and standard error of the mean (SEM) are shown. * *p* < 0.05; ** *p* < 0.01; *** *p* < 0.001; **** *p* < 0.0001.

Gene expression analysis of tRAS markers is illustrated in Figure 8. TNF-α-induced the gene expression of all tRAS markers. The strongest increases were seen in AGTR1, AGTR2, and ACE2. Notably, there was a time-dependent effect, particularly for AGTR1, with most of the increase seen following 24 h of exposure. AGTR1 inhibitor candesartan 100 µM significantly decreased TNF-α-induced expression of AGTR1, AGTR2, MAS1, and ACE2 after 4 but not after 24 h of exposure. In contrast, it increased ACE expression (100 µM) and decreased the expression of CTSD (1 µM and 100 µM) after 24 h of exposure. The AGTR2 receptor agonist CGP-42112A did not significantly affect the tRAS markers for up to 24 h of exposure in the concentration range examined. Furthermore, we found that the MAS1 receptor agonist AVE 0991 was able to decrease the expression of ACE, ACE2, and CTSD significantly compared to the TNF-α-only group after 24 h of exposure.

#### 3.3.3. Protein Validation

**Figure 9:**
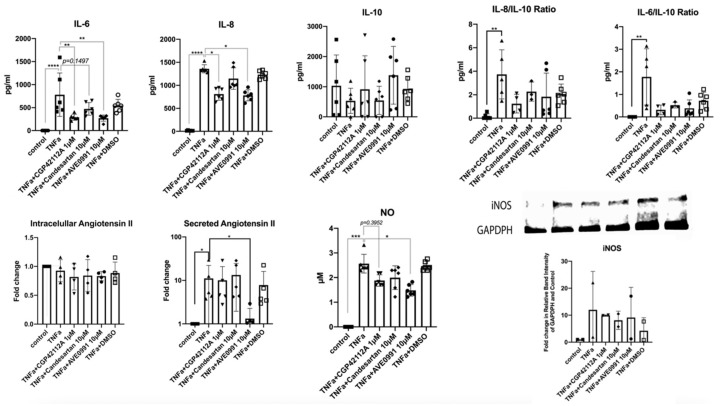
Protein alterations of IL-6, IL8, IL10, IL-8/IL-10 ratio, IL6-IL-10 ratio, as well as intracellular and secreted angiotensin II and iNOS in human nucleus pulposus cells after 24 h of exposure to candesartan (10 µM), the MAS-receptor agonist AVE 0991 (10 µM), and the AGTR2-receptor agonist CGP-42112A (1 µM) in inflammatory settings (TNF-α addition). DMSO (1 µL/mL) shown in µL volume on the *x*-axis) was used as the solvent control. Fold change values are shown. For the iNOS analysis, fold change in relative band intensity of GAPDPH and control are shown. *n* = 3 biological replicates and *n* = 2 technical replicates per donor were assessed in the ELISA analysis. *n* = 2 biological replicates were assessed for the western blot analysis. ImageJ was used for Western blot quantification [37]. Mean and standard deviations (SD) are shown.* *p* < 0.05; ** *p* < 0.01; *** *p* < 0.001; **** *p* < 0.0001.

Protein analysis for inflammatory markers, angiotensin II and iNOS, is shown in Figure 9. TNF-α was able to significantly increase the protein production of the IL-6, IL-8, IL-6/IL-10 ratio, and the IL-8/IL-10 ratio. MAS1 receptor agonist AVE 0991 (10 µM) and the AGTR2 receptor agonist CGP-42112A (1 µM) significantly reversed this effect, whereas the AGTR1 inhibitor candesartan (10 µM) effect was not significant. We did not find significant effects on intracellular angiotensin II concentrations for the drugs and TNF-α; still, there was a significant increase in angiotensin II secretion into the medium induced by TNF-α, which was reversed by the MAS1 receptor agonist AVE 0991 (10 µM). As seen before, TNF-α also induced NO_2_ production. This effect was reduced by the stimulation of the protective tRAS axis (AGTR2 and MAS1 agonists) but not by the inhibition of the AGTR1 receptor. Finally, iNOS production was induced by TNF-α but could not be reversed by the drugs.

## 4. Discussion

### 4.1. Main Findings

The present study aimed to evaluate the expression and functional role of tRAS in human NP cells. A particular focus was set on the impact of angiotensin II. Additionally, we were interested in investigating the effect of the inhibition of the tRAS axis (AGTR1) and the outcome of stimulation of the tRAS axis AGTR2, MAS1. There are mainly three primary findings we could unveil with the present experiments for the first time in humans: (1) tRAS components (including the SARS-CoV-2 entry receptor ACE2) exist on the gene and protein expression level in human NP cells; (2) the expression of tRAS markers are significantly associated with cells’ inflammatory and tissue degenerative state; (3) angiotensin II addition, the stimulation of the protective tRAS axis and the inhibition of the pathological tRAS axis are, at least partly, affecting the inflammatory and tissue degenerative state of NP cells.

### 4.2. Role of Angiotensin II in Human NP Cells

Our results should be evaluated in the context of two other workgroups that investigated the role of tRAS in IVD [29,40]. Morimoto et al. showed that tRAS components exist both in the AF and NP of rat IVDs [29]. Cell proliferation and the gene expression of tissue phenotype markers (such as COL1A1) could be mildly induced by angiotensin II addition. Although the authors did not assess inflammatory markers, their conclusion that angiotensin II had minor stimulatory effects on rat NP cells could be partly replicated in human NP cells. Our findings revealed that angiotensin II was able to induce NO_2_ production. Similar results were shown recently by Sun et al., who showed that angiotensin II induces ROS production in human NP cells [40]. In contrast to Morimoto et al., they showed that Ang II decreased cell viability and promoted cell senescence and apoptosis of NP cells. We could not confirm cell proliferative or anti-proliferative effects in our cell viability analysis. These controversial findings regarding cell proliferation/apoptosis might indicate that the impact of angiotensin II on cells might be related to the cell type and the receptor constitution, as indicated before [41]. Ang II can stimulate both tRAS axes and have paradoxical effects inducing both proliferation and apoptosis, depending on the cell type and receptor ratio of the pathological to the protective axis [11]. The AngII/ACE/AGTR1 axis promotes vasoconstriction, cell proliferation, inflammation, oxidative stress, hypertrophy, and fibrosis, whereas the alternative pathway, ACE2/Ang-(1–7)/MasR, counteracts these processes by inducing vasodilation, increase in cell coupling, decrease in cell volume and apoptosis [11].

### 4.3. ACE2 Is Expressed in Human NP Cells

Interestingly, our study also revealed the existence of angiotensin-converting enzyme 2 (ACE2) in human NP cells. ACE2 is expressed by cells of several tissues, which are usually highly perfused, such as enterocytes, renal tubules, gall bladder, cardiomyocytes, male reproductive cells, placental trophoblasts, ductal cells, eye, and vasculature [42]. Its existence in human NP cells is interesting as the IVD is known to be an immune-privileged organ that usually has no vascularization in the NP in healthy individuals [9]. However, as soon as inflammatory and degenerative processes are present, vascularization and innervation of the whole IVD, including the NP, can be found [5,7]. ACE2 is the entry receptor of SARS-CoV-2, a virus responsible for the coronavirus disease 2019 (COVID-19) [43]. Considering the fact that numerous publications indicate musculoskeletal problems and low back pain in patients affected by COVID-19 [15,16,17,18] and that degeneration of the IVD leads to “access” to the vasculature raises the question of whether SARS-CoV-2 is also found in IVD and might target the ACE2 available here. Hence, it would be interesting to evaluate whether IVD degeneration might predict musculoskeletal outcomes in COVID-19 infections. Indeed, Bakılan et al. reported that back pain during acute COVID-19 infections was significantly related to post-COVID-19 musculoskeletal symptoms [18] indicating that potential degenerative conditions predestine for a worse outcome in affected patients. Džubera et al. recently showed that surgical treatment of IVD improved the pain and neurological status in selected cases with COVID-19 suffering from vertebral pain [17]. As low back pain highly affects life quality in patients, further examination of the role of COVID-19 and ACE2 in low back pain patients is warranted.

### 4.4. tRAS Expression Is Correlated with the Inflammatory State of NP Cells

**Figure 10:**
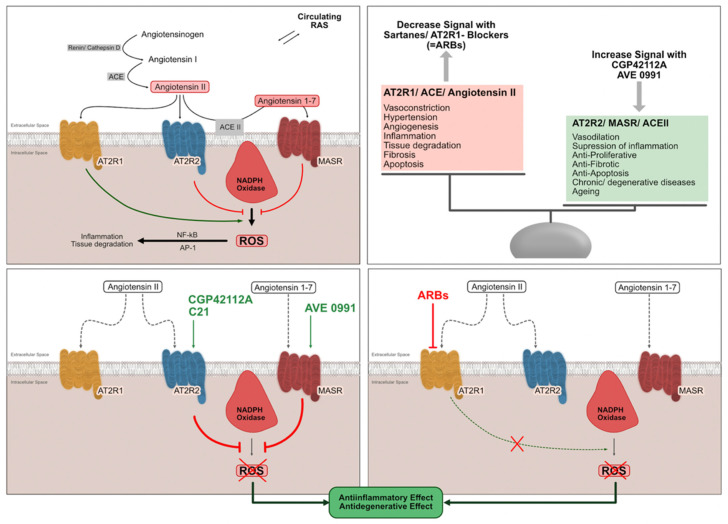
Illustration of tissue renin-angiotensin system (tRAS) mechanisms in humans [11]. Local renin-angiotensin systems are found in human nucleus pulposus cells. The angiotensin II receptor type 2 (AGTR2) and the Ang1–7/Mas receptor (MasR) axis counteract the pro-inflammatory effects (increasing intracellular reactive oxygen species (ROS) of accumulating angiotensin II concentrations (AngII) on angiotensin II receptor type 1 (AGTR1). The inhibition of the pathological axis (AGTR1) and the stimulation of the protective axis could show beneficial effects on the inflammatory and degenerative state of cells. ARBs: angiotensin II type 1 receptor blockers.

In accordance with the finding that the tRAS expression was highly correlated with the inflammatory state of the cells, the inhibition of the pathological tRAS (AGTR1) axis and the stimulation of the protective axis (AGTR2, MAS1) revealed beneficial effects on inflammation and degeneration in human NP cells. As there is no evidence for comparisons for NP cells yet, external validation of these results is warranted. However, the anti-inflammatory and anti-degenerative effects of AVE0991 (MAS1 receptor agonist) [36], CGP-42112A [13,35] and candesartan [33,34] were already shown in other tissues, which revealed similar effects as demonstrated in our study. In accordance with our results, AVE0991 was able to significantly reduce the secretion of Ang II and NO_2_ into the medium of endothelial cells and smooth muscle cells, as also shown by other authors [44,45]. However, NO_2_ release was also shown to be induced by AVE0991 in some cell types and receptor expression characteristics, indicating that the present findings cannot be fully generalized for other tissues/cell types [46]. Notably, the MAS1, AGTR1, and AGTR2 receptors are known to interact with each other physically; this might potentially contribute to the balancing of the tRAS expression state of cells depending on different cell conditions as proposed by the current evidence [11,13,47]. Thus, the pharmaceutical balancing (i.e., modulation of tRAS receptor activation levels through inhibition and stimulation) of the tRAS was recently introduced as a novel therapeutic approach in inflammatory and degenerative diseases [11,13,48]. The potential mechanisms of the tRAS based on the current evidence and the present findings are shown in Figure 10.

### 4.5. Modulation of tRAS as a Potential Target in Intervertebral Disc Disease

Notably, Sun et al. also found that the degenerated IVD tissue showed activated pathological tRAS components (e.g., AGTR1, ACE), as also shown in our group before [30,40]. They also showed that the degree of IVD degeneration was correlated with the expression of the pathological tRAS axis. The inhibition of the pathological tRAS axis and the stimulation of the protective tRAS axis showed beneficial effects in the present study, supporting findings published for other cell types [11]. AGTR1 inhibition via another AGTR1-antagonist (losartan) also revealed beneficial effects on human NP cells in a previous study of our group, although some of the effects of losartan were related to additional pathways [31]. Sun et al. also revealed that the pathological tRAS axis accelerated IVD degeneration and aging in a mouse model, potentially through the Nrf2/NF-κB signal cascade [40]. We could not find evidence that iNOS was involved in the reaction pathways as inhibition of AGTR1 and stimulation of the protective axis (AGTR2/MAS1) did not influence iNOS protein production, as shown in the mouse model in the study of Sun et al. [40]. However, this could be due to the fact that the authors used a mouse model of spontaneously hypertensive rats (SHRs), which could have affected the reactions and receptor expressions of the local renin-angiotensin system in vivo. The authors also mentioned this limitation, concluding that SHRs show overactivated systematic RAS. Thus, the authors’ provided results might differ from those in the non-hypertensive situation. An increase in Ang II levels regulates both inducible nitric oxide synthase (iNOS) and endothelial nitric oxide synthase (eNOS) in a different manner according to results from different cell types [49]. Amplification of mRNA and protein levels of eNOS by the peptide is associated with an increase in nitrite production in cultured bovine pulmonary artery endothelial cells [50]. Calcium-dependent eNOS has been demonstrated to stimulate NO release via AT1 receptors as a result of increased Ang II levels [51]. Acute Ang II causes a reduction in renal haemodynamics, which is associated with an increase in renal eNOS mRNA expression, and long-term Ang II levels increase eNOS protein levels [52]. It is also known that Ang II inhibits iNOS expression through its AT1 receptor, which is a well-established inhibitor of iNOS. However, this was mainly shown for endothelial cells and vascular smooth muscle cells (VCMCs). Based on the findings of Nakayama et al. [53], Ang II inhibits iNOS, which, in turn, disrupts the production of NO by cultured VSMCs as a result of the cytokine-induced inhibition of NO production. Furthermore, in rat astroglial cultures, the AT1 receptors reduce iNOS mRNA and protein levels [54]. In addition, in intact isolated vessels, Ang II produced by the endothelium inhibits iNOS expression [55]. Overall, our results indicate that, at least for human nucleus pulposus cells, the effects might not be mediated via iNOS. Other evidence for iNOS alterations following tRAS modulation in human nucleus pulposus cells is currently not available for result comparisons. Notably, our results showed that drug effects were time-dependent. Time plays a critical role in the action of drugs. There are many factors that influence the scheduling of drugs, including the duration of inhibition of the target or the residence time of the drug molecule on the target. Some targets can be intermittently blocked and still be effective therapeutic targets. It is not possible to determine the reasons for the different time dependencies of the drugs chosen based on our experiments. Our results were meant to provide the first insight into tRAS modulation with different drugs. An in-depth drug sensitivity analysis would be required to determine the best concentrations and exposure times for human NP cells for each drug. However, our results can be used for further studies exploring the effects of these drugs on human NP cells limiting the experiments to the time points with the maximum effects.

### 4.6. Strengths and Limitations

The present study is associated with strengths and limitations. To the best of our knowledge, it is the first study confirming the existence of the tRAS components in human NP cells on both the gene and protein levels. It also provides novel insight into the inhibition of the pathological tRAS axis and the stimulation of the protective tRAS axis in humans. Furthermore, the experimental design included a multiperspective approach focusing on angiotensin II effects, tRAS modulation, and its associations with inflammation and degeneration on the gene expression and protein level. There are implications for future research and clinical practice based on the present findings. The findings could serve as a fundament for future studies examining the intracellular pathways of tRAS. A major problem with current therapeutic options is that they do not address the pathophysiological roots of IVD degeneration. In the context of tRAS modulation therapy, the drugs should be considered as affecting both the systemically available components of the RAS, their effects on the vasculature, their interaction with the tRAS systems in human tissues, and the local tRAS components when these drugs reach tissue levels. In a recent study, we demonstrated that the inflammatory environment of human nucleus pulposus cells requires high concentrations of ARBs to be inhibited. By using such cell culture studies, we are able to gain knowledge about the locally available tRAS without the influence of systemic effects and to emphasize the possibility of an independent system (at least in part). It is worth noting that these concentrations of RAS inhibitors are probably not achievable with usual oral dosages and systemic availability of these drugs in humans, which may also explain why studies have not found the relevance of these drug classes in COVID-19. In the future, as new drug delivery methods emerge, it may be possible to deliver effective doses of these drugs to specific tissues of interest and block the overactive pathological tRAS where necessary. Due to the fact that research on the protective tRAS pathway only began a few years ago in the work of Unger et al., there is a limited amount of information available on the involved mechanisms. In order to prepare for human studies involving these tRAS modulation drugs, animal in vivo experiments could be performed in the next step. As a result, intervertebral disc disease could be treated with a non-surgical strategy targeted at restoring the balance of tRAS in the diseased disc. Limitations mainly concern the potential clinical implementation. There is no evidence regarding the concentrations reached in the IVD after oral application. Thus, the present findings might not be translatable to future in vivo findings. Novel application techniques and targeted approaches limiting the systemic effects of tRAS modulation drugs might quicken clinical applications. There are already some tRAS modulation drugs used for COVID-19 and other diseases showing promising anti-inflammatory effects. [11,13,56]. Furthermore, more in-depth analyses, including knockdown studies of the tRAS components and increased expression of Ang II, are warranted to examine the intracellular pathways of tRAS. Particularly, the role of the mitochondrion and the nucleus in the tRAS pathways should be further investigated, as these structures are known to be highly relevant for the tRAS effects [11]. Some promising findings on the role of the mitochondrion in tRAS pathways were recently investigated by Sun et al. in the IVD and highlight the importance of the pathways in macrophage infiltration, inflammation, oxidative cell, and cell senescence [11]. Notably, the discs used in the present study were obtained from donors with Pfirrmann grade 2–3; 30–37-year–old males. Our previous research examined the gene expression of tRAS components in different pathologies. Obtaining healthy tissue for research is difficult because most human disc issues are obtained from diseased patients. Previously, our group identified gene expression of tRAS components in a representative patient cohort (mostly male) that included a wide range of indications (infection, degeneration, trauma, and scoliosis) and localizations (cervical, thoracic, and lumbar spines) for discectomy and spinal fusion. AGT, matrix-metalloproteinases 13 and 3, IL-1, IL-6, and IL-8 were more abundant in traumatic IVDs compared to degenerated IVDs in tRAS-positive samples. Further research is required to determine whether there is a difference in the expression of tRAS components between healthy and degenerated discs. 

## 5. Conclusions

The present study provided novel insights into the tissue-renin-angiotensin system (tRAS) in human NP cells. The findings showed that the major components of tRAS found in highly perfused tissues are also available in human NP cells and are associated with inflammatory processes. The modulation of the tRAS was able to alter the cells’ inflammatory and degenerative/phenotype state. The findings have several relevant implications for future research. The intracellular pathways of tRAS, the role of ACE2 and COVID-19 in IVD, and the application of the tRAS modulation drugs in vivo should be further investigated. These efforts could open the door for a novel therapeutic approach for patients suffering from IDD and low back pain.

## Figures and Tables

**Table 1:** Study design.

Study Group	Medium Composition	Supplier Reference Number	Manufacturer
Control	Basal Medium	-	-
Angiotensin II	Basal Medium + Angiotensin II: 0.1 µM, 10 µM	A9525-5MG	Sigma Aldrich
TNF-α	Basal Medium + 10 ng/mL TNF-α	210-TA-020	R&D Systems Europe, Ltd.
TNF-α + Angiotensin II	Basal Medium + TNF-α: 10 ng/mL + Angiotensin II: 0.1 µM, 10 µM	TNF-α: 210-TA-020 Angiotensin II: A9525-5MG	TNF-α: R&D Systems Europe, Ltd. Angiotensin II: Sigma Aldrich
TNF-α + CGP42112A	Basal Medium + TNF-α: 10 ng/mL + CGP42112A: 0.1 µM, 1 µM, 10 µM	TNF-α: 210-TA-020 CGP42112A: C160-1MG	TNF-α: R&D Systems Europe, Ltd. CGP42112A: Sigma Aldrich
TNF-α + AVE 0991	Basal Medium + TNF-α: 10 ng/mL + AVE 0991: 1 µM, 10 µM, 100 µM	TNF-α: 210-TA-020 AVE 0991: HY-15778-5MG	TNF-α: R&D Systems Europe, Ltd. AVE 0991: Lucerna-Chem
TNF-α + Candesartan	Basal Medium + TNF-α: 10 ng/mL + Candesartan: 1 µM, 10 µM, 100 µM	TNF-α: 210-TA-020 Candesartan: SML0245-10MG	TNF-α: R&D Systems Europe, Ltd. CGP42112A: Sigma Aldrich
TNF-α + DMSO	Basal Medium + Solvent control: DMSO (0.1 µL/mL, 1 µL/mL, 10 µL/mL)	-	-

**Table 2:** Description of primary antibodies.

Primary Antibody	Dilution	Supplier Reference Number	Manufacturer
AGTR1 Polyclonal Antibody	1:125	PA5-20812	Invitrogen
AGTR2 Polyclonal Antibody	1:125	PA3-210	Invitrogen
ACE2 Polyclonal Antibody	1:100	PA5-20040	Invitrogen
MAS1 Polyclonal Antibody	1:100	PA5-77282	Invitrogen

**Table 3:** Characteristics of custom-designed primer-probes and gene expression assays (Applied Biosystems (Waltham, MA, USA)) used for gene expression analysis.

Gene Acronym	Gene Full Name	Primer Sequence or Catalogue Number	Reporter/Quencher
*hRPLP0*	Human 60S acidic ribosomal protein P0	Forward seq.: 5′-TGG GCA AGA ACA CCA TGA TG-3′ Reverse primer seq.: 5′-CGG ATA TGA GGC AGC AGT TTC-3′	FAM/TAMRA
*hACAN*	Human aggrecan	Forward seq.: 5′-AGT CTT CAA GCC TCC TGT ACT CA-3′ Reverse primer seq.: 5′-CGG GAA GTG GCG GTA ACA-3′	FAM/TAMRA
	Human angiotensinogen	Hs01586213_m1	
*hACE*	Human angiotensin-converting enzyme	Hs00174179_m1	FAM/NFQ-MGB
*hAGTR1a*	Human angiotensin-II receptor type 1	Hs00258938_m1	FAM/NFQ-MGB
*hAGTR2*	Human angiotensin-II receptor type 2	Hs02621316_s1	FAM/NFQ-MGB
*hMAS1*	Human MAS1 proto-oncogene, G protein-coupled receptor	Hs00267157_s1	FAM/NFQ-MGB
*hCTSD*	Human cathepsin D	Hs00157205_m1	FAM/NFQ-MGB
*hCOL1A1*	Human collagen type 1 alpha 1 chain	Forward seq.: 5′-CCC TGG AAA GAA TGG AGA TGA T-3′ Reverse primer seq.: 5′-ACT GAA ACC TCT GTG TCC CTT CA-’3	FAM/TAMRA
*hCOL2A1*	Human collagen type 2 alpha 1 chain	Forward seq.: 5′-GGC AAT AGC AGG TTC ACG TAC A-3′ Reverse primer seq.: 5′-GAT AAC AGT CTT GCC CCA CTT ACC-3′	FAM/TAMRA
*hIL6*	Human interleukin 6	Hs00985639_m1	FAM/NFQ-MGB
*hIL8*	Human interleukin 8	Hs00174103_m1	FAM/NFQ-MGB
*hMMP1*	Human matrix-metalloproteinase 1	Hs00899568_m1	FAM/NFQ-MGB
*hMMP3*	Human matrix-metalloproteinase 3	Hs00968305_m1	FAM/NFQ-MGB
*hIL1-b*	Human interleukin 1b	Hs00174097_m1	FAM/NFQ-MGB
*hTLR4*	Human toll-like receptor 4	Hs00152939_m1	FAM/NFQ-MGB
*hTNFα*	Human tumor necrosis factor *α*	Hs00174128_m1	FAM/NFQ-MGB

## Data Availability

Datasets are available on request. The raw data and all related documents supporting the conclusions of this manuscript will be made available by the authors, without undue reservation, to any qualified researcher.

## Supplementary Materials

The following supporting information can be downloaded at: https://www.mdpi.com/article/10.3390/cells11213418/s1. Figure S1: Gene expression analysis in human NP cells for angiotensin II (0.1 µM (“ATlow”) and 10 µM (“AThigh”) with 24 h of exposure; Figure S2: Gene expression analysis in human NP cells for angiotensin II (0.1 µM (“ATlow”) and 10 µM (“AThigh”) with 48 h of exposure; Figure S3: Gene expression analysis in human NP cells for Candesartan (1 µM, 10 µM, 100 µM), the MAS-receptor agonist AVE 0991 (1 µM, 10 µM, 100 µM), and the AGTR2-receptor agonist CGP-42112A (0.1 µM, 1 µM, 10 µM) with 4 h of exposure in inflammatory settings (TNF-α addition); Figure S4: Gene expression analysis in human NP cells for Candesartan (1 µM, 10 µM, 100 µM), the MAS-receptor agonist AVE 0991 (1 µM, 10 µM, 100 µM), and the AGTR2-receptor agonist CGP-42112A (0.1 µM, 1 µM, 10 µM) with 24 h of exposure in inflammatory settings (TNF-α addition).
