# Supplementary material for: In Vitro Characterization of a Tissue Renin-Angiotensin System in Human Nucleus Pulposus Cells
Source: Cells. 2022 Oct 28;11(21):3418. doi: 10.3390/cells11213418 (PMC9656476; doi:10.3390/cells11213418)
Supplement: Supplementary file 1 [file cells-11-03418-s001.zip › Figure S2.pdf]

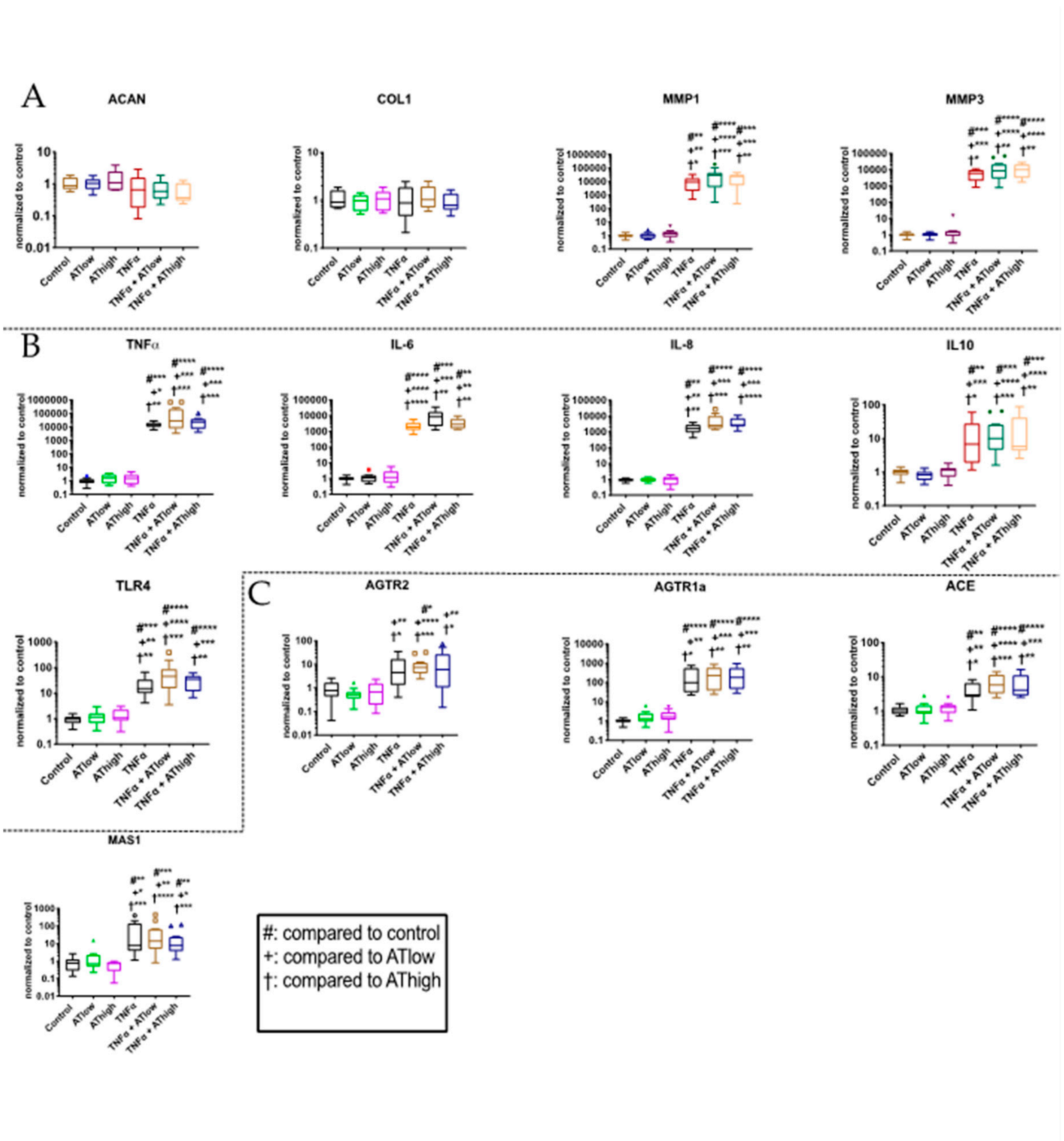

Supplementary Figure S2. Gene expression analysis in human NP cells for angiotensin II (0.1μM (“ATlow”) and 10μM (“AThigh) with 48 h of exposure. Fold change values obtained from the 2^(-DeltaDeltaCt) method and normalized to the baseline (day 0) are shown. n=4 biological replicates and n=3 technical replicates per donor were assessed. Box plots are shown (median and interquartile range). Outliers are represented as symbols. #: compared to control; +: compared to ATlow (0.1μM); †: compared to AThigh (10μM). * $p < 0.05$; ** $p < 0.01$; *** $p < 0.001$; **** $p < 0.0001$.
