# Supplementary material for: In Vitro Characterization of a Tissue Renin-Angiotensin System in Human Nucleus Pulposus Cells
Source: Cells. 2022 Oct 28;11(21):3418. doi: 10.3390/cells11213418 (PMC9656476; doi:10.3390/cells11213418)
Supplement: Supplementary file 1 [file cells-11-03418-s001.zip › Figure S4.pdf]

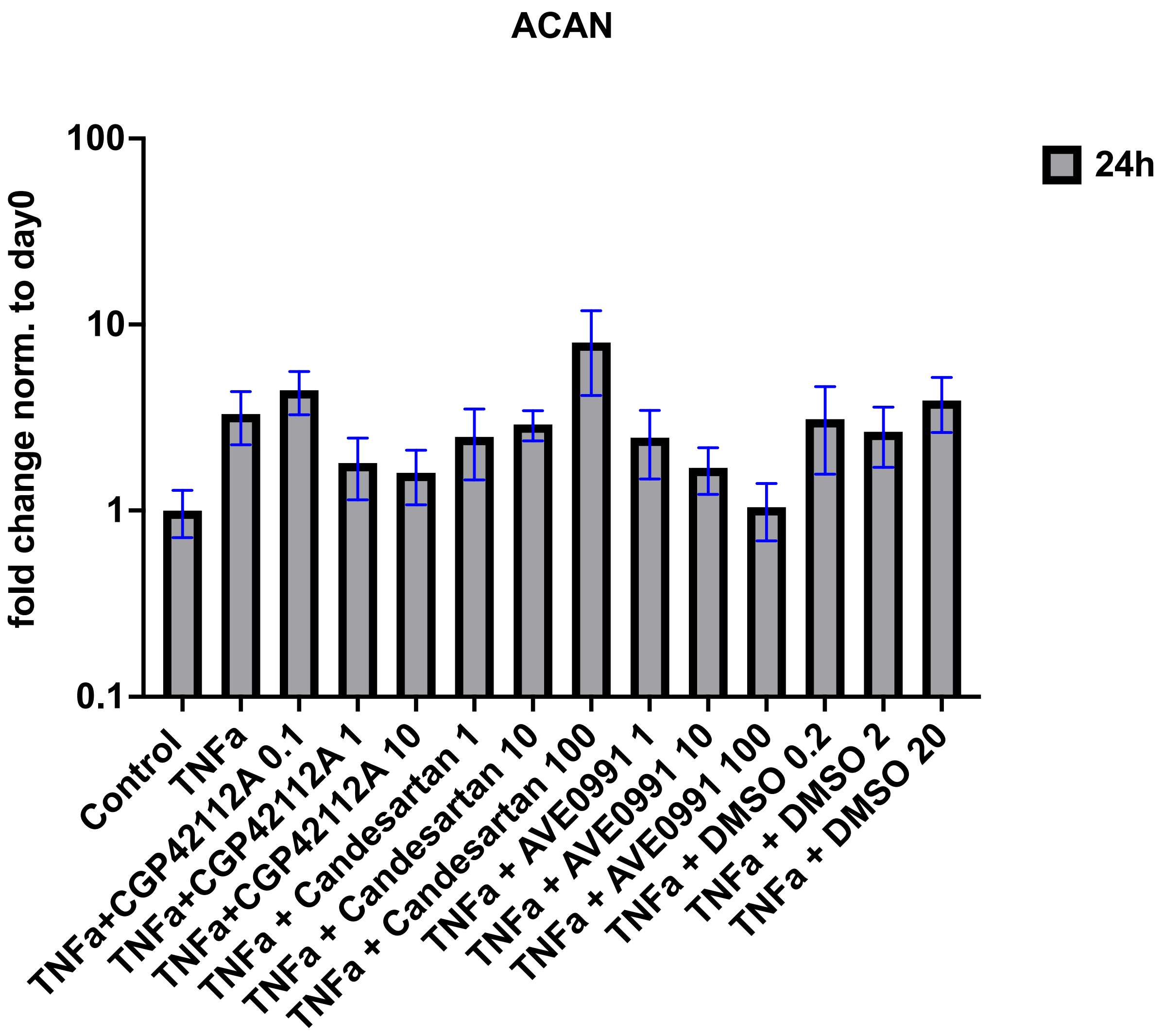
ACAN
fold change norm. to day0
100
10
1
0.1
24h
Control
TNFa
TNFa+CGP42112A 0.1
TNFa+CGP42112A 1
TNFa+CGP42112A 10
TNFa + Candesartan 1
TNFa + Candesartan 10
TNFa + Candesartan 100
TNFa + AVE0991 1
TNFa + AVE0991 10
TNFa + AVE0991 100
TNFa + DMSO 0.2
TNFa + DMSO 2
TNFa + DMSO 20

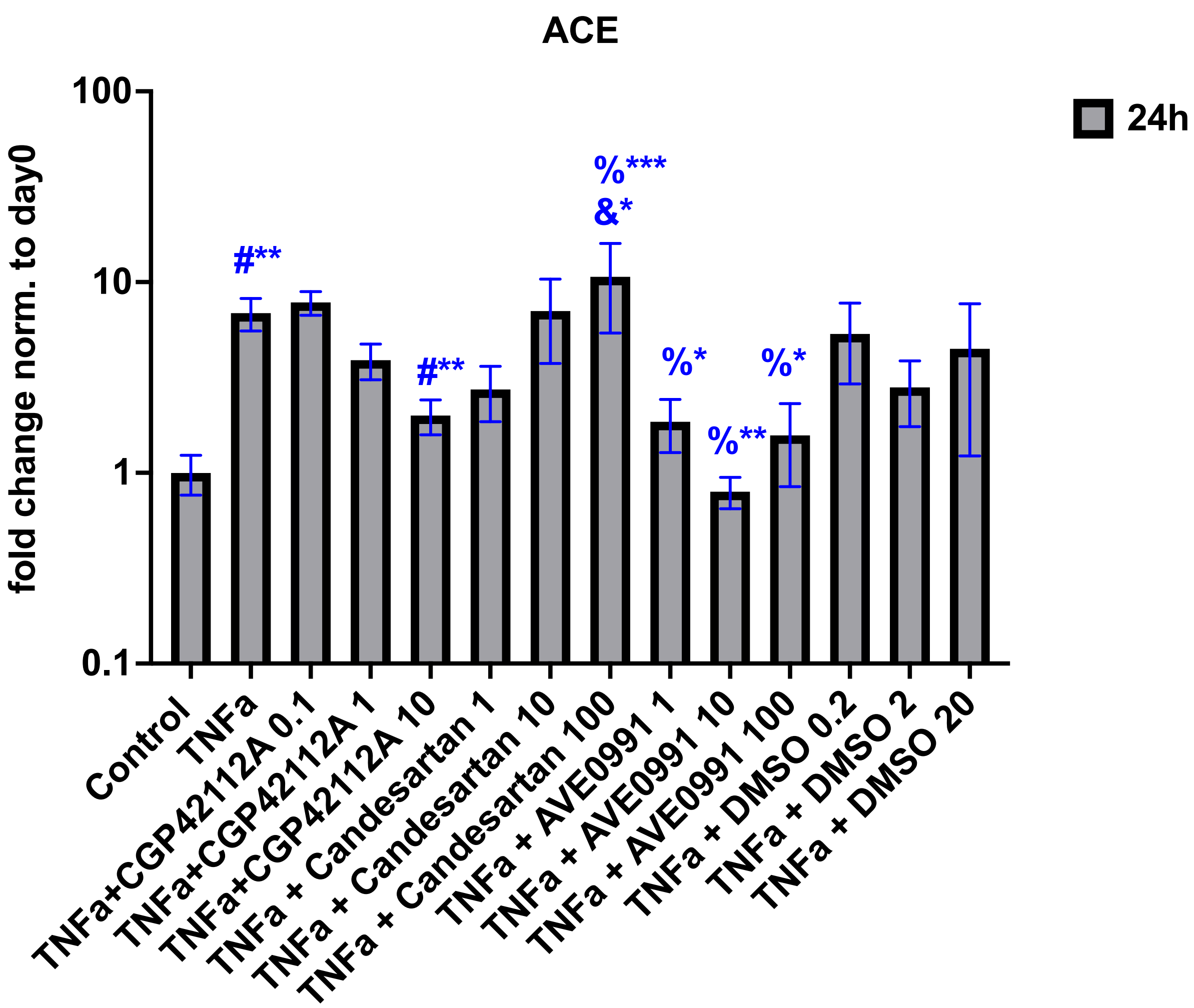
ACE
fold change norm. to day0
100
10
1
0.1
24h
#**
#**
%***
&*
%*
%**
%*
Control
TNFa
TNFa+CGP42112A 0.1
TNFa+CGP42112A 1
TNFa+CGP42112A 10
TNFa + Candesartan 1
TNFa + Candesartan 10
TNFa + Candesartan 100
TNFa + AVE0991 1
TNFa + AVE0991 10
TNFa + AVE0991 100
TNFa + DMSO 0.2
TNFa + DMSO 2
TNFa + DMSO 20

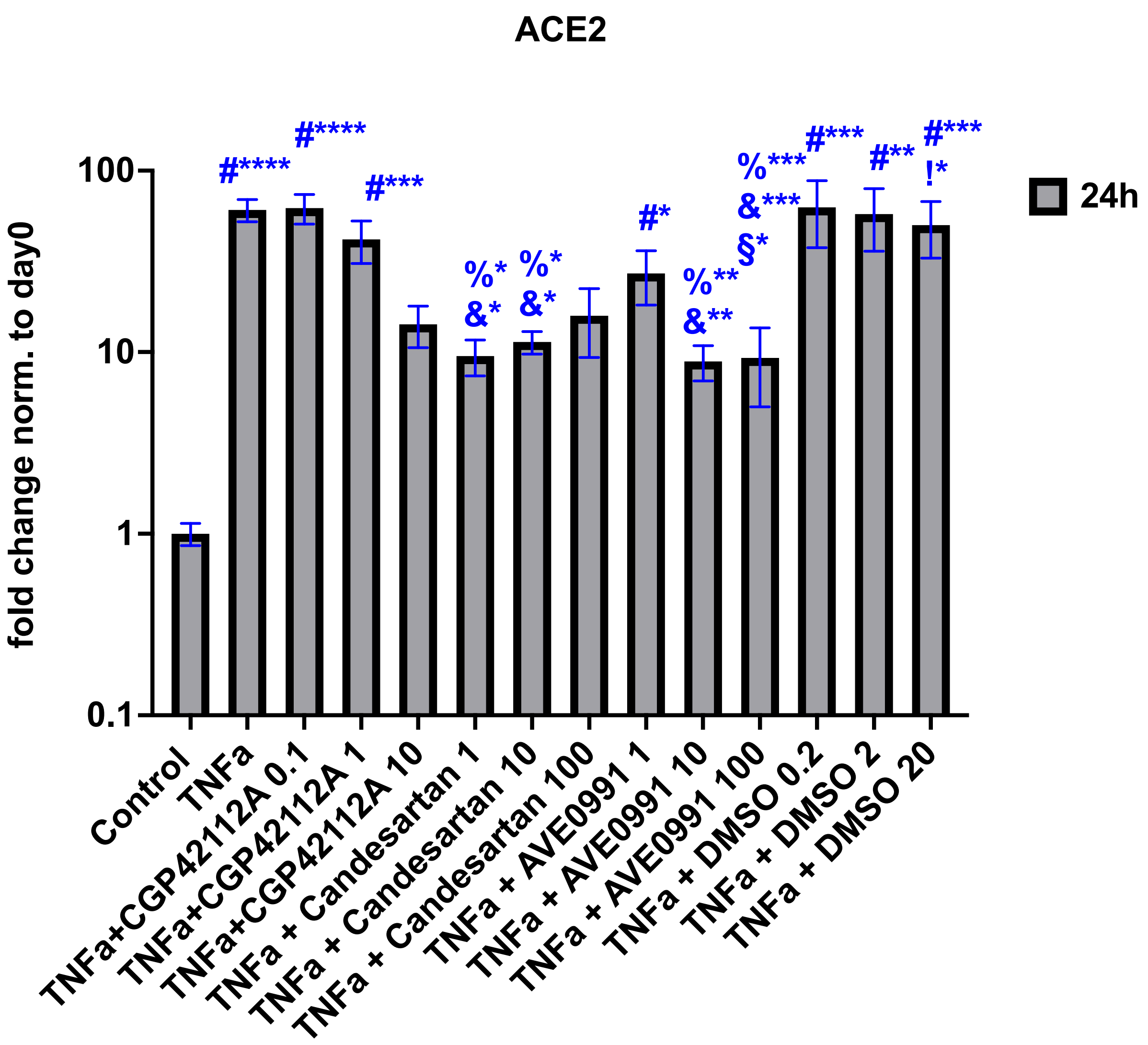
ACE2
fold change norm. to day0
100
10
1
0.1
24h
#****
#****
#***
%*
&*
%*
&*
#*
%**
&**
%***
&***
§*
#***
#**
#***
!*
Control
TNFa
TNFa+CGP42112A 0.1
TNFa+CGP42112A 1
TNFa+CGP42112A 10
TNFa + Candesartan 1
TNFa + Candesartan 10
TNFa + Candesartan 100
TNFa + AVE0991 1
TNFa + AVE0991 10
TNFa + AVE0991 100
TNFa + DMSO 0.2
TNFa + DMSO 2
TNFa + DMSO 20

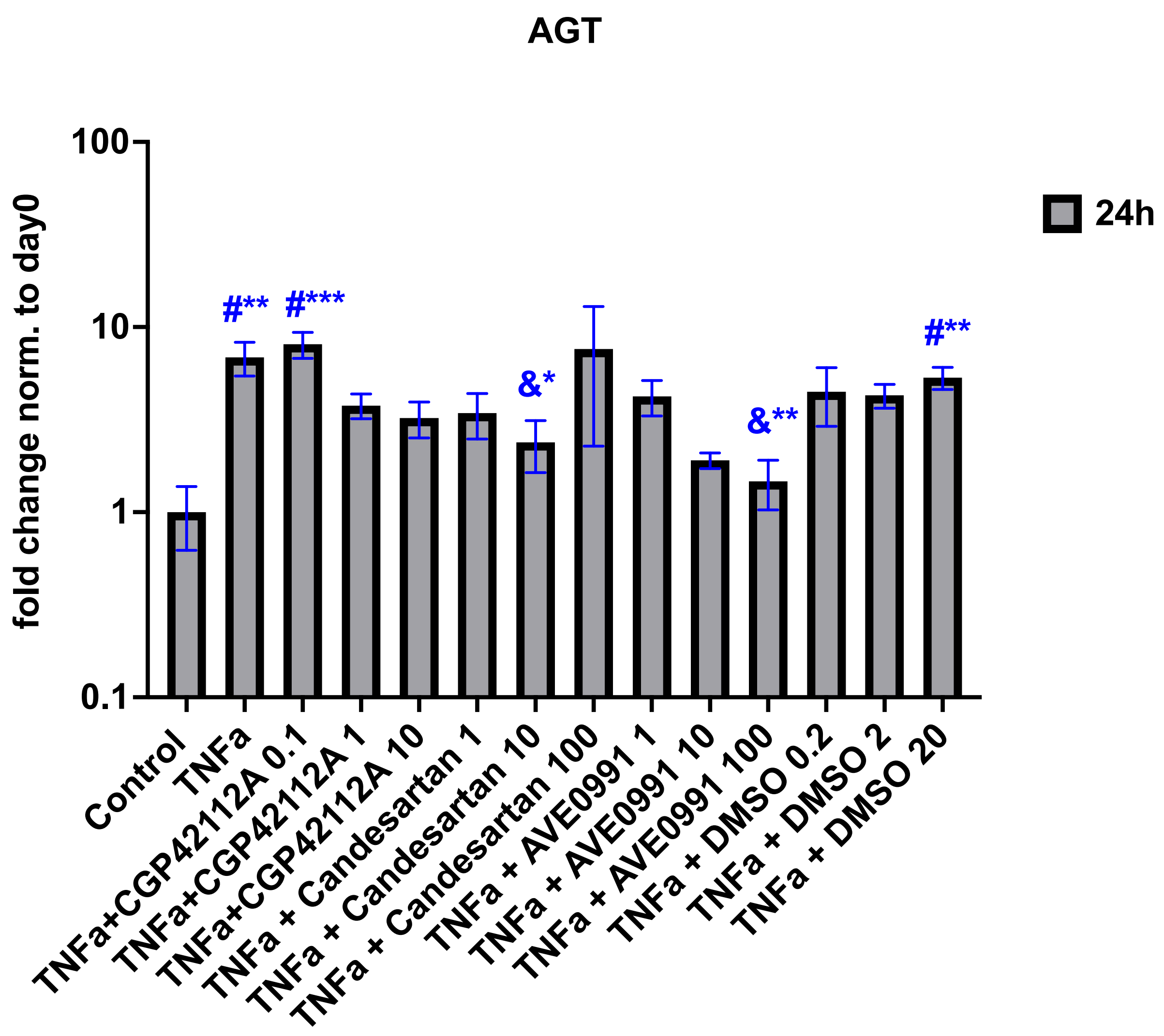

AGT
fold change norm. to day0
100
10
1
0.1
24h
#**
#***
&*
&**
#**
Control
TNFa
TNFa+CGP42112A 0.1
TNFa+CGP42112A 1
TNFa+CGP42112A 10
TNFa + Candesartan 1
TNFa + Candesartan 10
TNFa + Candesartan 100
TNFa + AVE0991 1
TNFa + AVE0991 10
TNFa + AVE0991 100
TNFa + DMSO 0.2
TNFa + DMSO 2
TNFa + DMSO 20

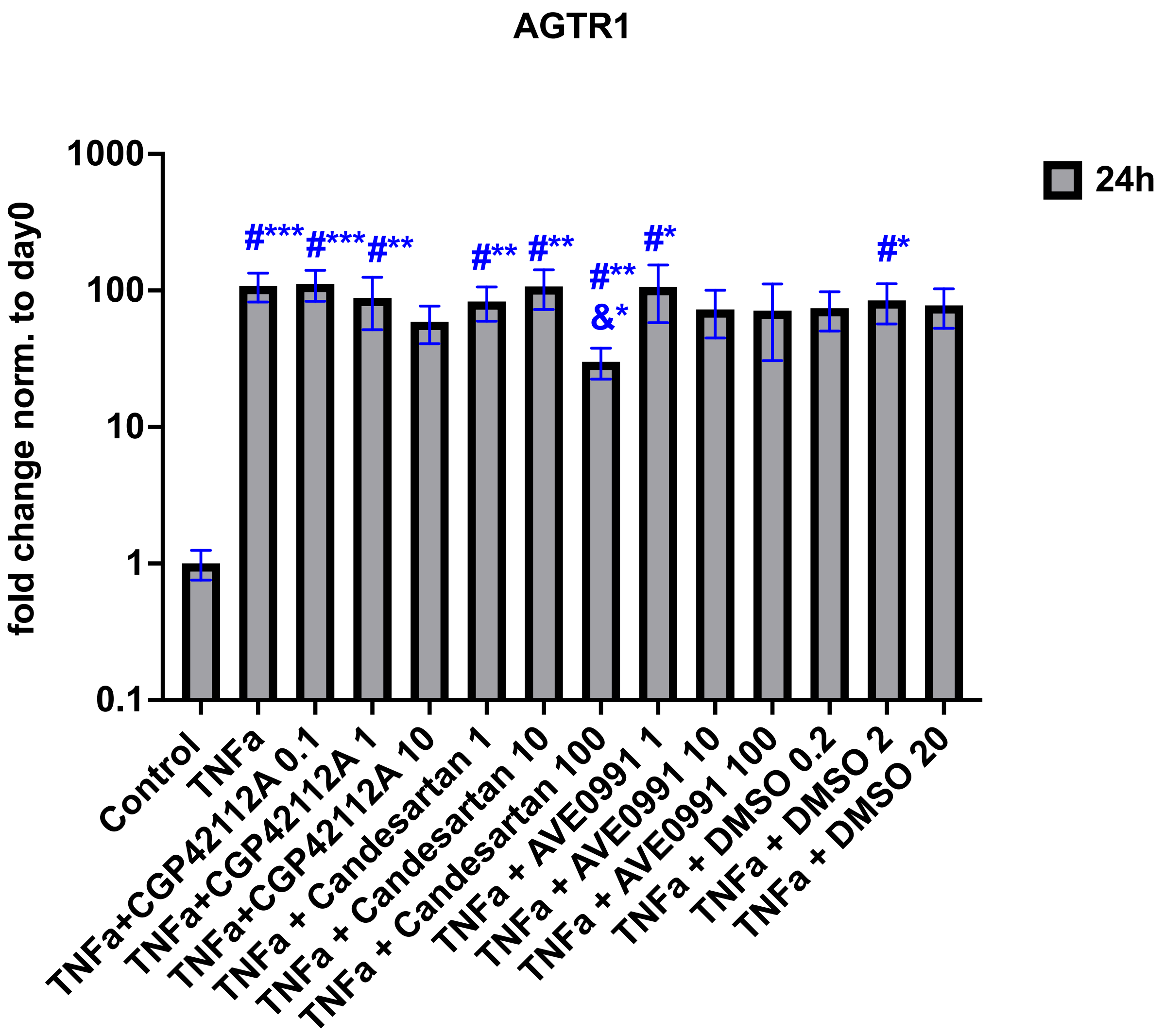
AGTR1
24h
fold change norm. to day0
1000
100
10
1
0.1
#***
#***
#**
#**
#**
#**
&*
#*
#*
Control
TNFa
TNFa+CGP42112A 0.1
TNFa+CGP42112A 1
TNFa+CGP42112A 10
TNFa + Candesartan 1
TNFa + Candesartan 10
TNFa + Candesartan 100
TNFa + AVE0991 1
TNFa + AVE0991 10
TNFa + AVE0991 100
TNFa + DMSO 0.2
TNFa + DMSO 2
TNFa + DMSO 20

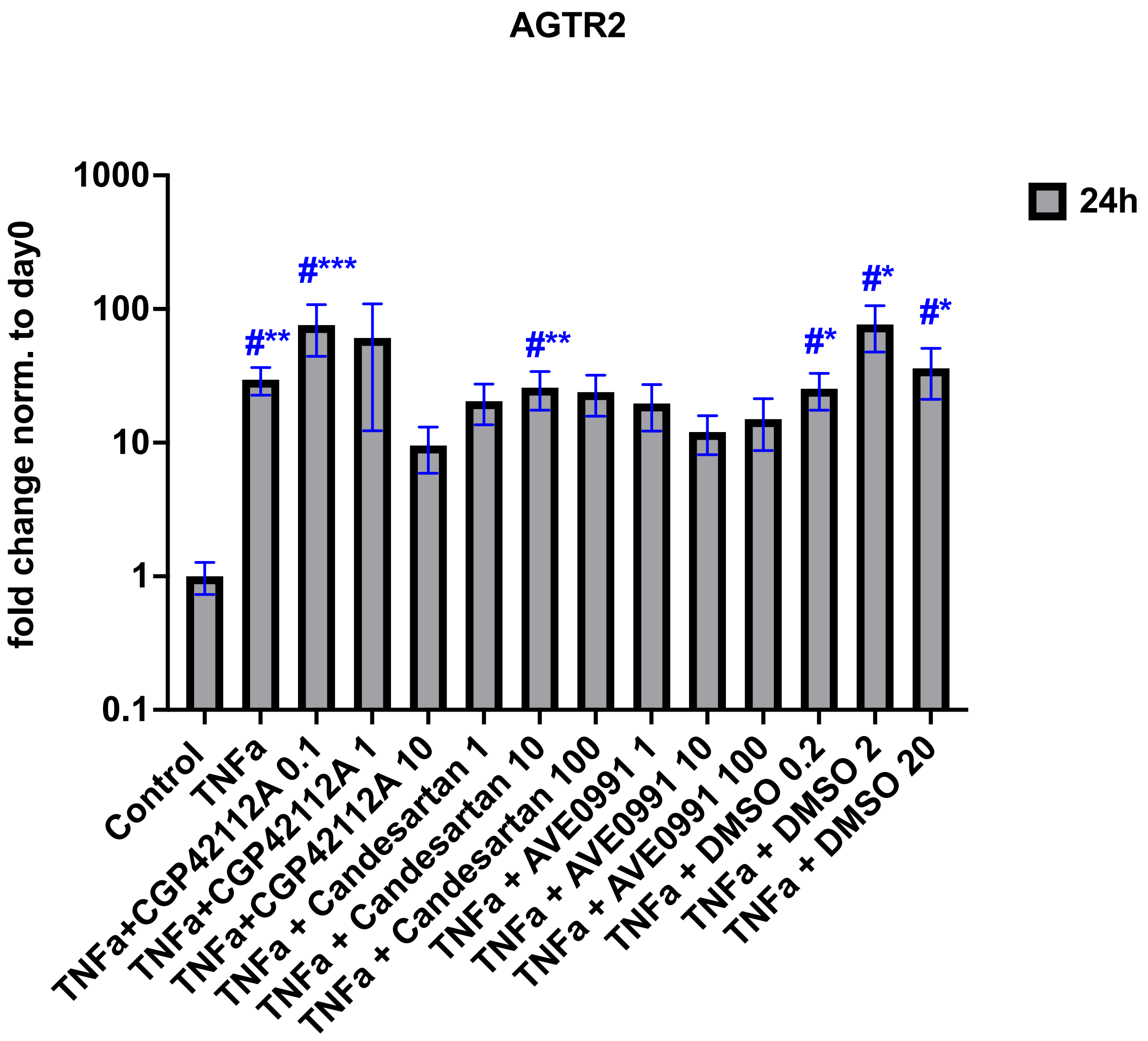
AGTR2
fold change norm. to day0
1000
100
10
1
0.1
24h
#**
#***
#**
#*
#*
#*
Control
TNFa
TNFa+CGP42112A 0.1
TNFa+CGP42112A 1
TNFa+CGP42112A 10
TNFa + Candesartan 1
TNFa + Candesartan 10
TNFa + Candesartan 100
TNFa + AVE0991 1
TNFa + AVE0991 10
TNFa + AVE0991 100
TNFa + DMSO 0.2
TNFa + DMSO 2
TNFa + DMSO 20

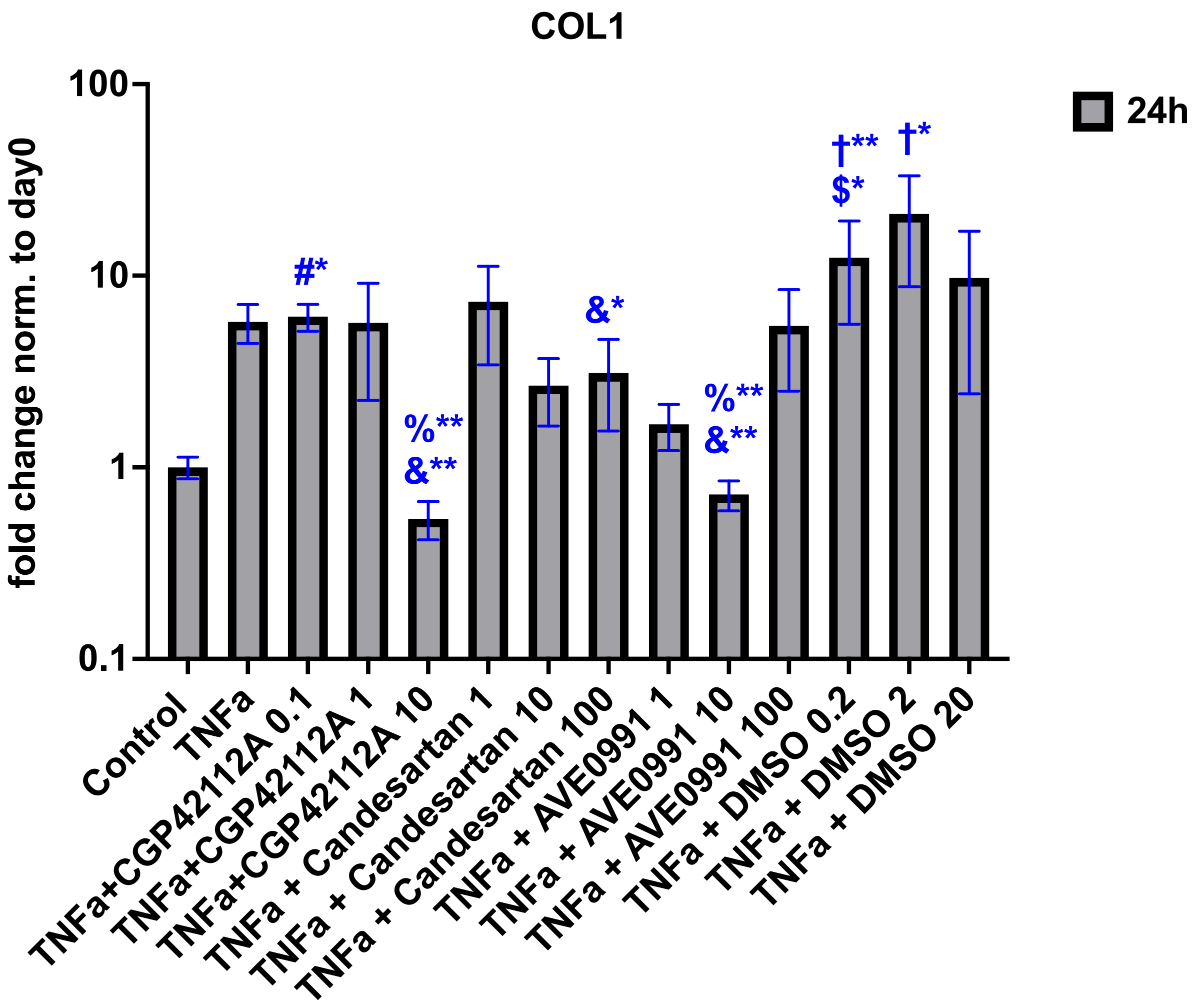
COL1
fold change norm. to day0
100
10
1
0.1
24h
#*
%**
&**
&*
%**
&**
†**
$*
†*
Control
TNFa
TNFa+CGP42112A 0.1
TNFa+CGP42112A 1
TNFa+CGP42112A 10
TNFa + Candesartan 1
TNFa + Candesartan 10
TNFa + Candesartan 100
TNFa + AVE0991 1
TNFa + AVE0991 10
TNFa + AVE0991 100
TNFa + DMSO 0.2
TNFa + DMSO 2
TNFa + DMSO 20

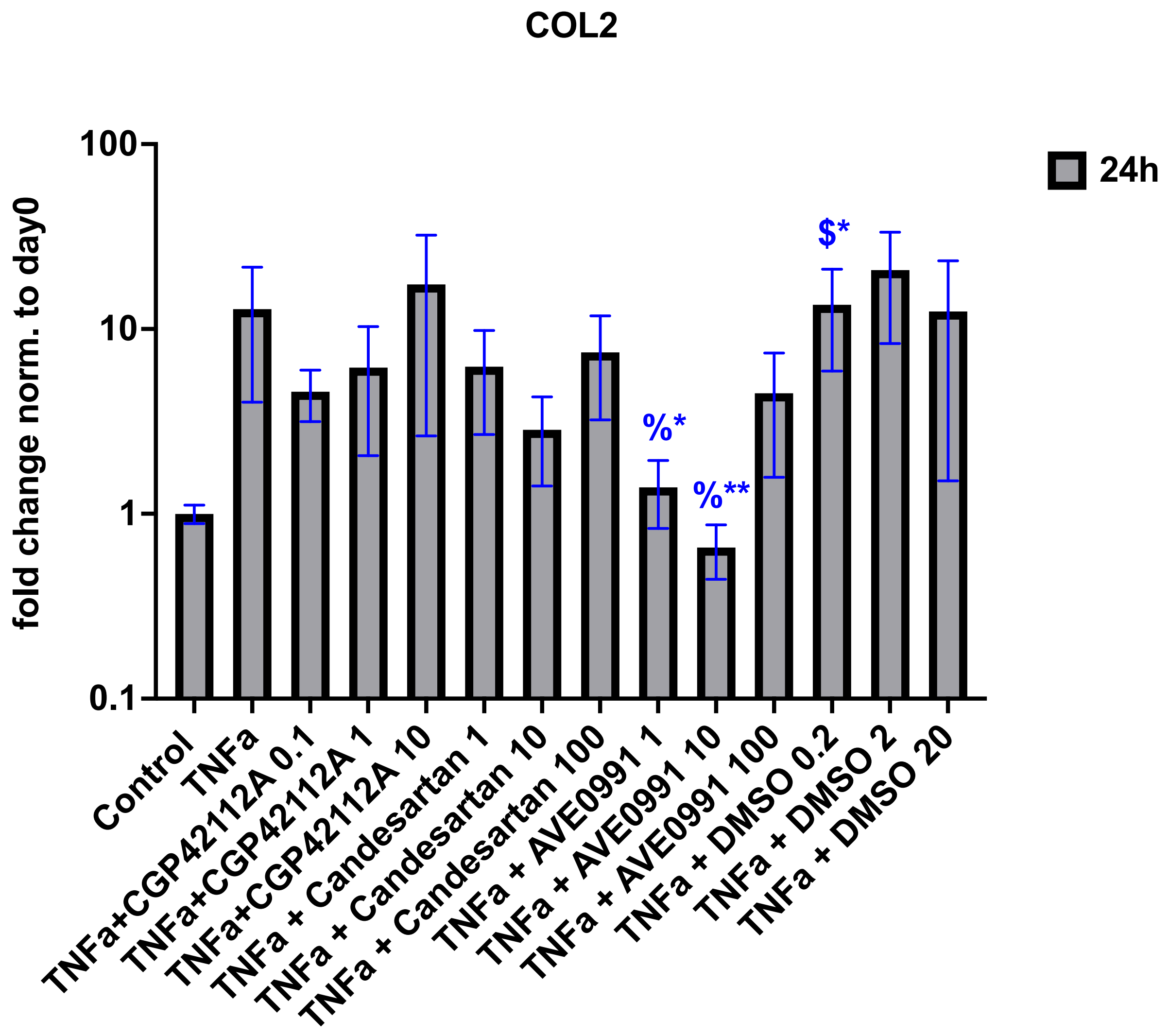
COL2
24h
fold change norm. to day0
100
10
1
0.1
$*
%*
%**
Control
TNFa
TNFa+CGP42112A 0.1
TNFa+CGP42112A 1
TNFa+CGP42112A 10
TNFa + Candesartan 1
TNFa + Candesartan 10
TNFa + Candesartan 100
TNFa + AVE0991 1
TNFa + AVE0991 10
TNFa + AVE0991 100
TNFa + DMSO 0.2
TNFa + DMSO 2
TNFa + DMSO 20

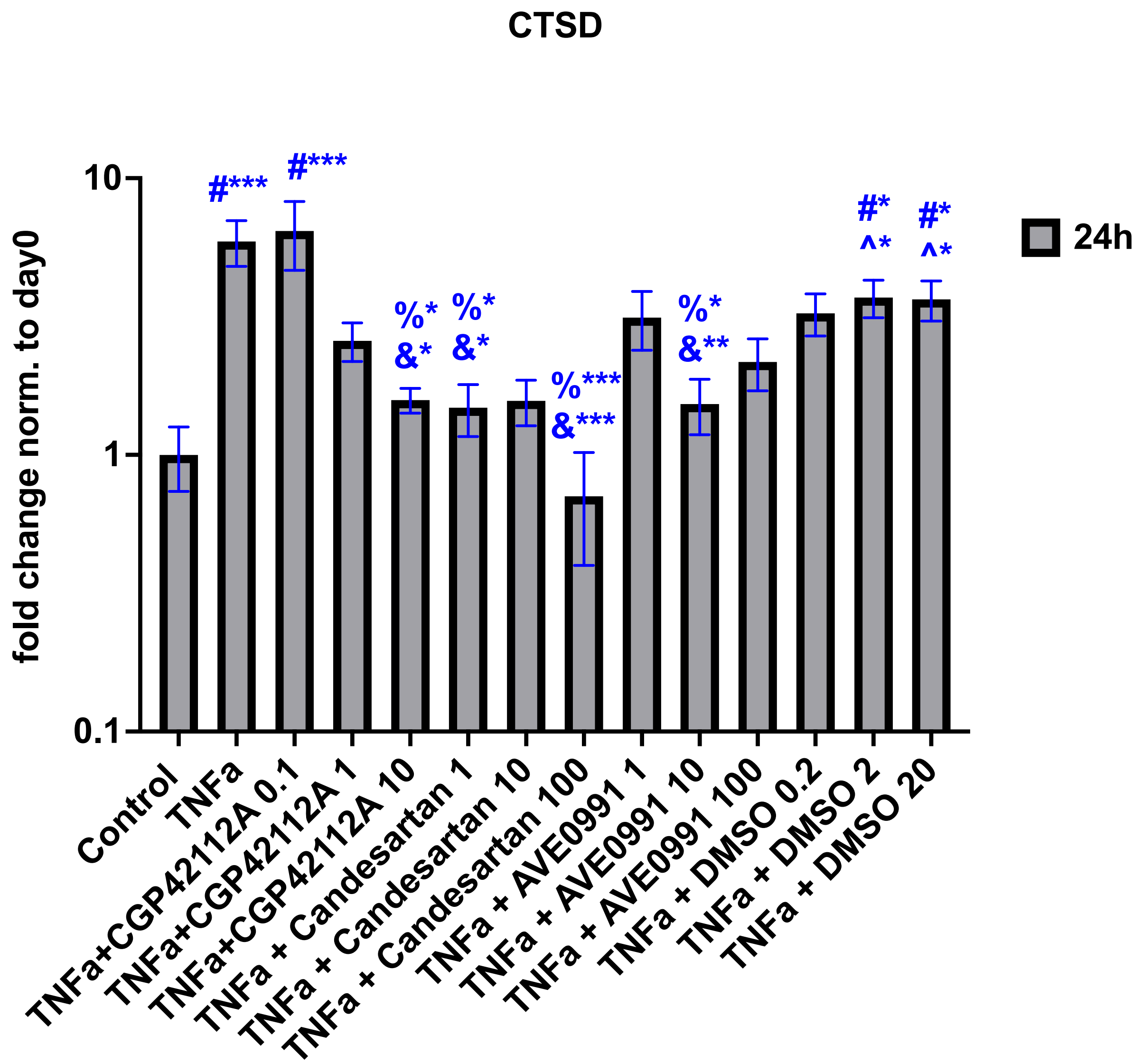
CTSD
fold change norm. to day0
10
1
0.1
24h
#***
#***
%*
&*
%*
&*
%***
&***
%*
&**
#*
^*
#*
^*
Control
TNFa
TNFa+CGP42112A 0.1
TNFa+CGP42112A 1
TNFa+CGP42112A 10
TNFa + Candesartan 1
TNFa + Candesartan 10
TNFa + Candesartan 100
TNFa + AVE0991 1
TNFa + AVE0991 10
TNFa + AVE0991 100
TNFa + DMSO 0.2
TNFa + DMSO 2
TNFa + DMSO 20

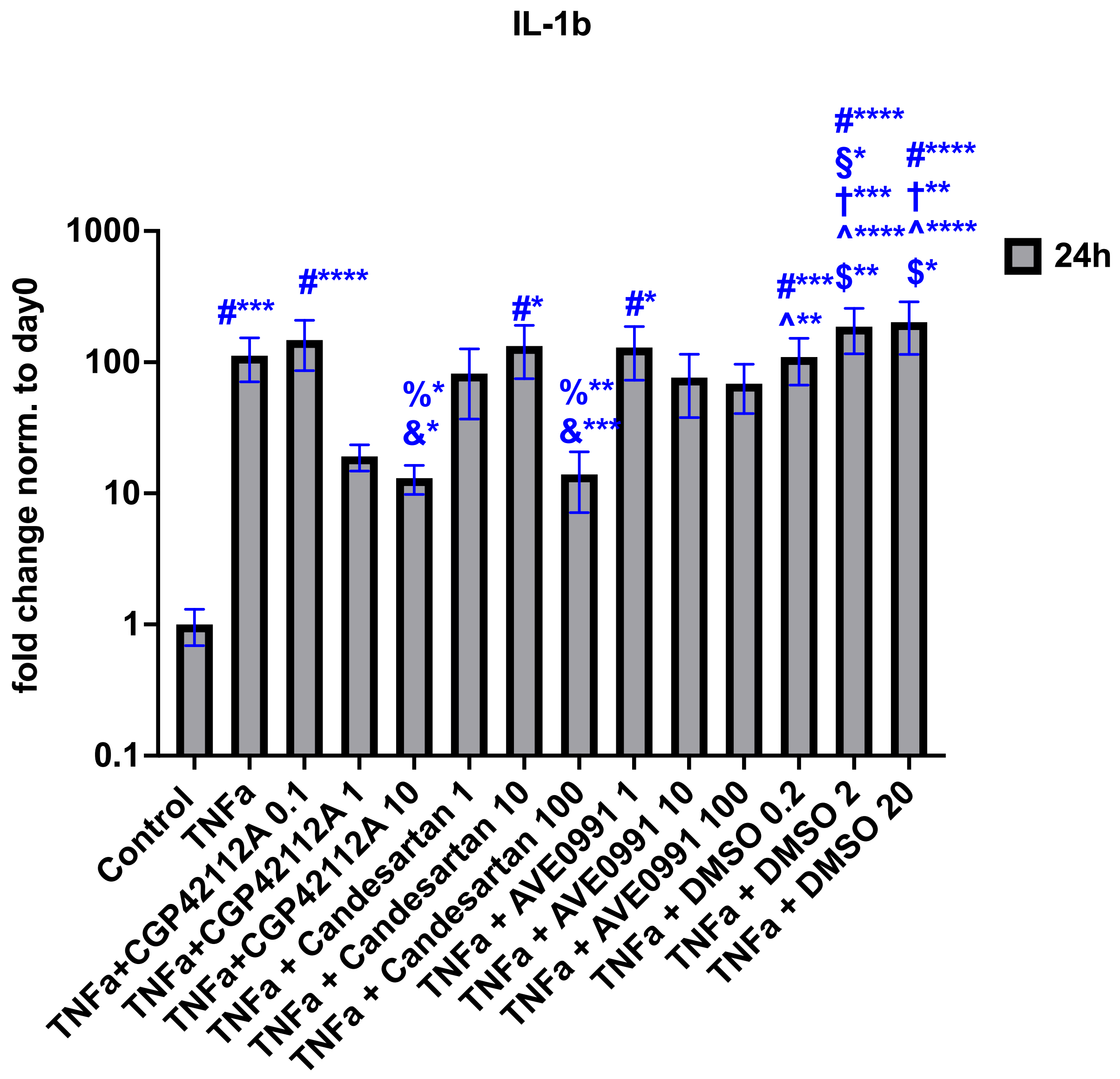
IL-1b
fold change norm. to day0
1000
100
10
1
0.1
24h
#***
#****
%*
&*
#*
%**
&***
#*
#***
^**
#****
§*
†***
^****
$**
#****
†**
^****
$*
Control
TNFa
TNFa+CGP42112A 0.1
TNFa+CGP42112A 1
TNFa+CGP42112A 10
TNFa + Candesartan 1
TNFa + Candesartan 10
TNFa + Candesartan 100
TNFa + AVE0991 1
TNFa + AVE0991 10
TNFa + AVE0991 100
TNFa + DMSO 0.2
TNFa + DMSO 2
TNFa + DMSO 20

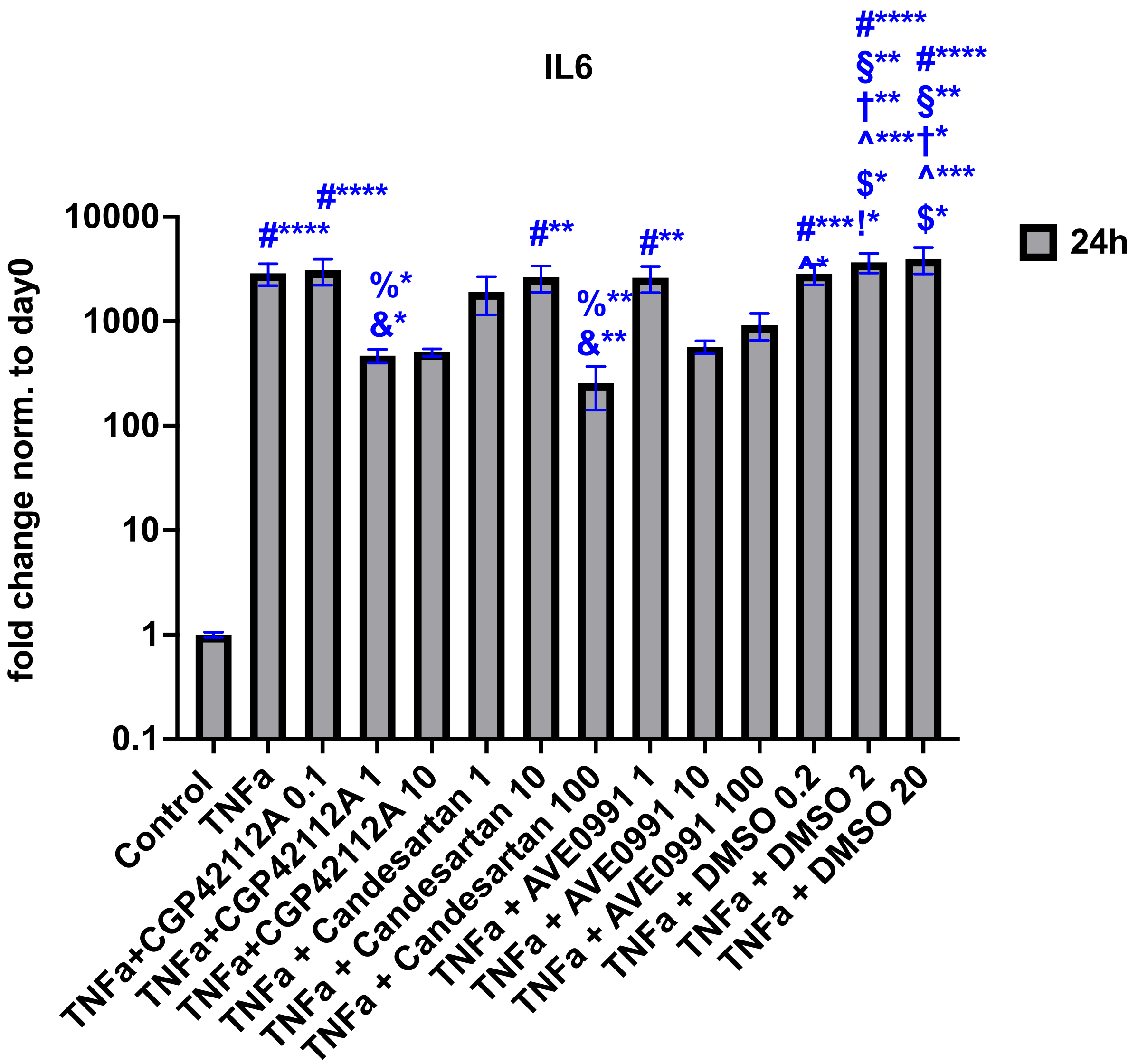
IL6
fold change norm. to day0
10000
1000
100
10
1
0.1
24h
#****
#****
%*
&*
#**
%**
&**
#**
#***
^*
#****
§**
†**
^***
$*
!*
#****
§**
†*
^***
$*
Control
TNFa
TNFa+CGP42112A 0.1
TNFa+CGP42112A 1
TNFa+CGP42112A 10
TNFa + Candesartan 1
TNFa + Candesartan 10
TNFa + Candesartan 100
TNFa + AVE0991 1
TNFa + AVE0991 10
TNFa + AVE0991 100
TNFa + DMSO 0.2
TNFa + DMSO 2
TNFa + DMSO 20

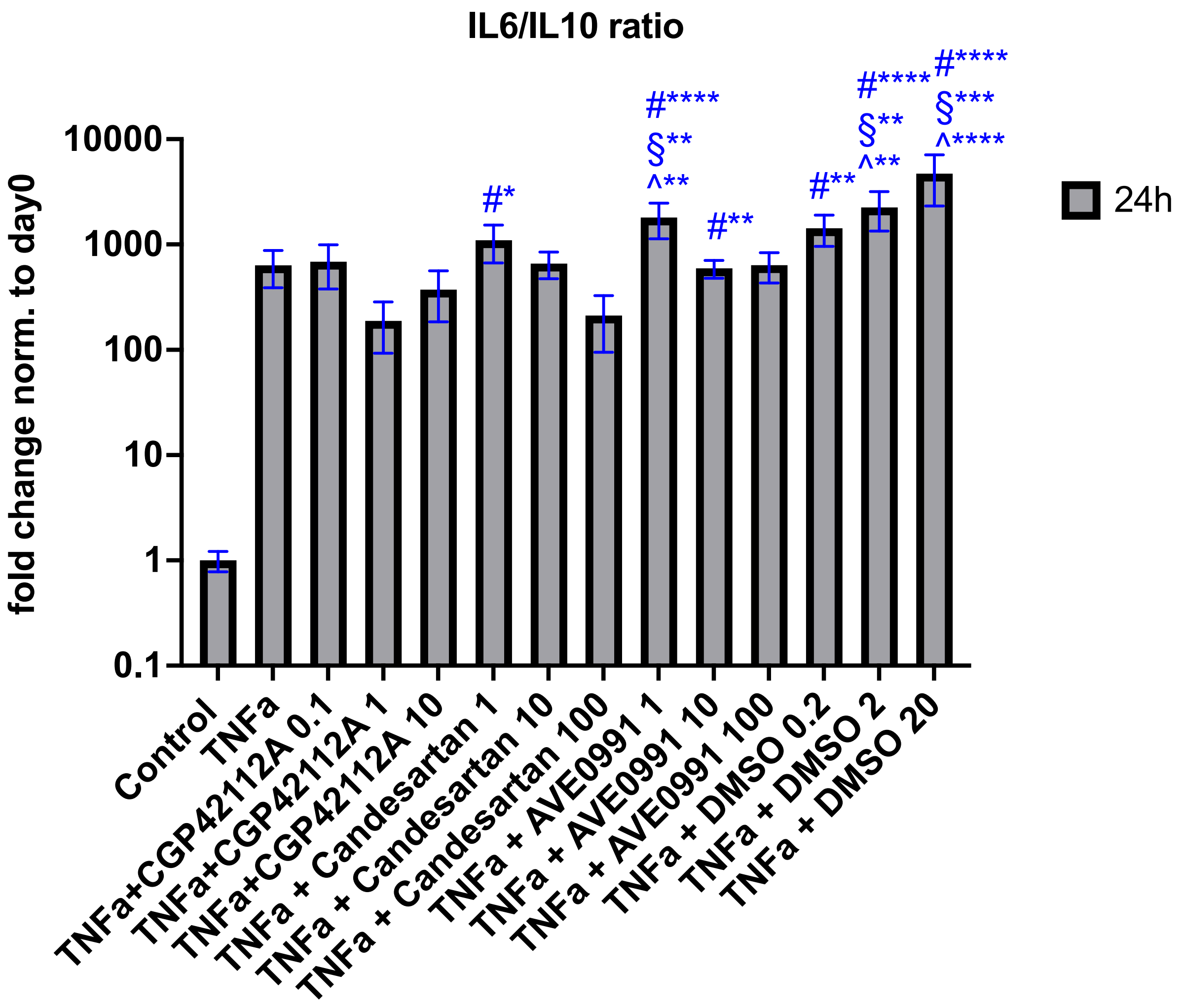
IL6/IL10 ratio
fold change norm. to day0
10000
1000
100
10
1
0.1
24h
#*
#****
§**
^**
#**
#**
#****
§**
^**
#****
§***
^****
Control
TNFa
TNFa+CGP42112A 0.1
TNFa+CGP42112A 1
TNFa+CGP42112A 10
TNFa + Candesartan 1
TNFa + Candesartan 10
TNFa + Candesartan 100
TNFa + AVE0991 1
TNFa + AVE0991 10
TNFa + AVE0991 100
TNFa + DMSO 0.2
TNFa + DMSO 2
TNFa + DMSO 20

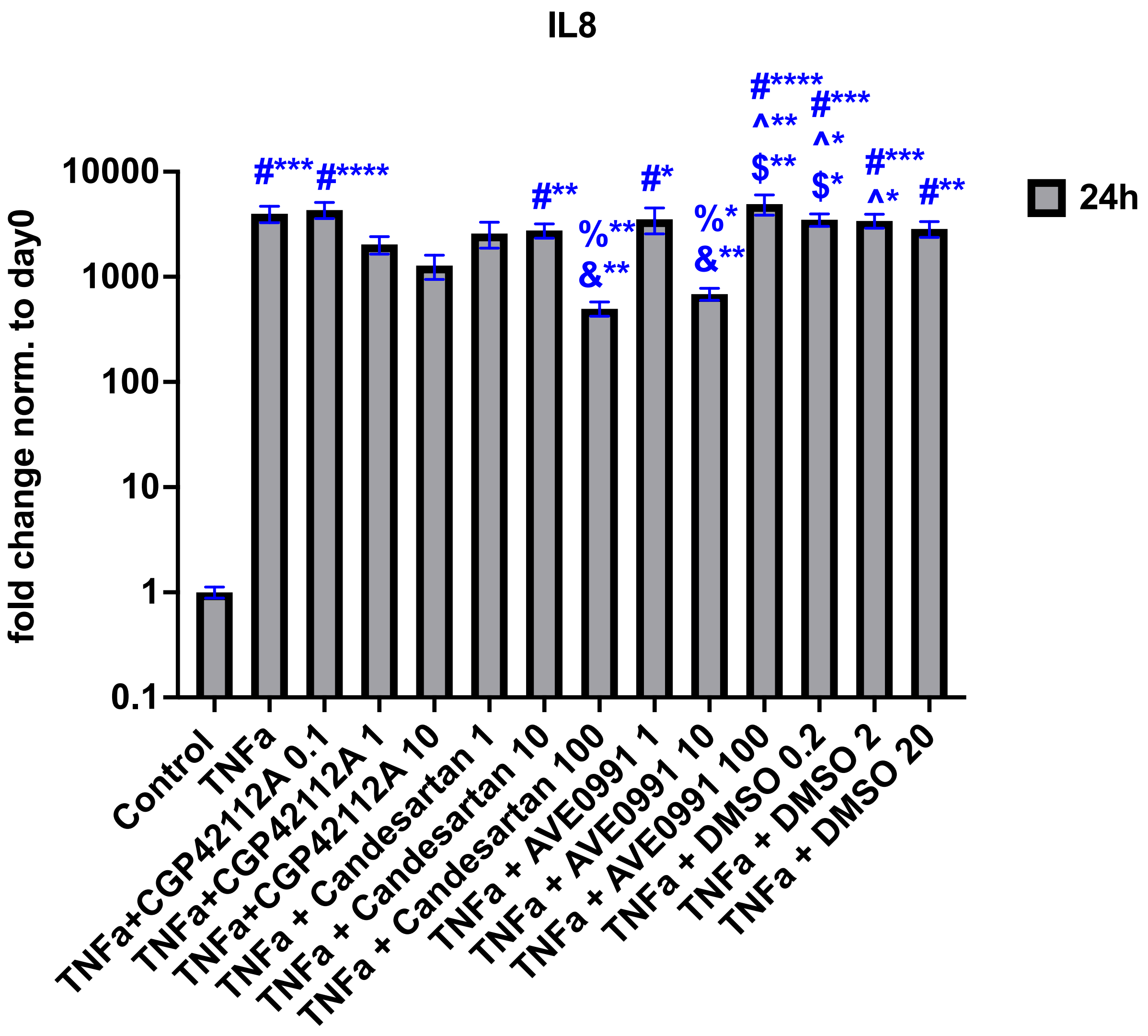

IL8
fold change norm. to day0
10000
1000
100
10
1
0.1
24h
#***
#****
#**
%**
&**
#*
%*
&**
#****
^**
$**
#***
^*
$*
#***
^*
#**
Control
TNFa
TNFa+CGP42112A 0.1
TNFa+CGP42112A 1
TNFa+CGP42112A 10
TNFa + Candesartan 1
TNFa + Candesartan 10
TNFa + Candesartan 100
TNFa + AVE0991 1
TNFa + AVE0991 10
TNFa + AVE0991 100
TNFa + DMSO 0.2
TNFa + DMSO 2
TNFa + DMSO 20

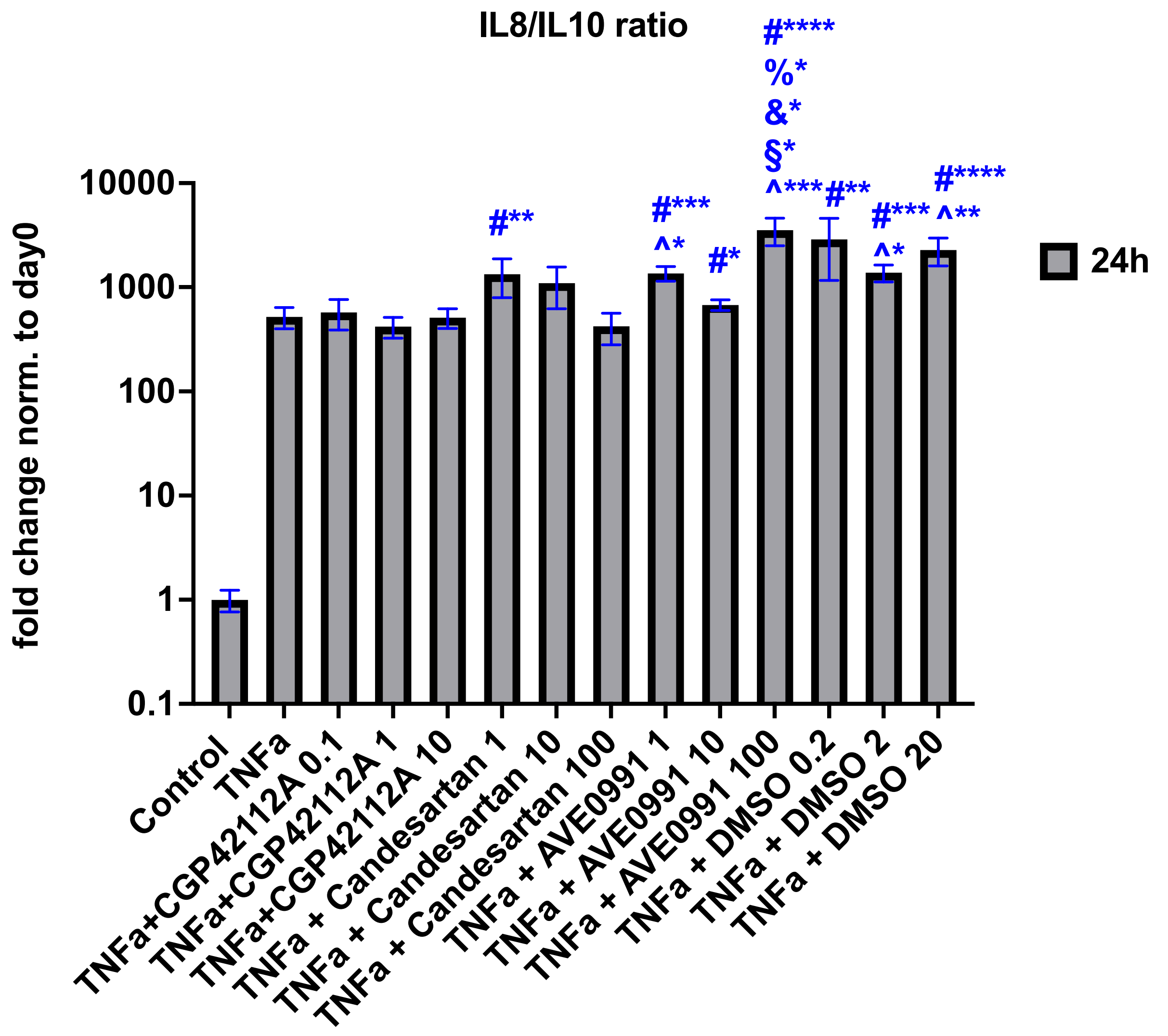

IL8/IL10 ratio
fold change norm. to day0
10000
1000
100
10
1
0.1
24h
#**
#***
^*
#*
#****
%*
&*
§*
^***
#**
#***
^*
#****
^**
Control
TNFa
TNFa+CGP42112A 0.1
TNFa+CGP42112A 1
TNFa+CGP42112A 10
TNFa + Candesartan 1
TNFa + Candesartan 10
TNFa + Candesartan 100
TNFa + AVE0991 1
TNFa + AVE0991 10
TNFa + AVE0991 100
TNFa + DMSO 0.2
TNFa + DMSO 2
TNFa + DMSO 20

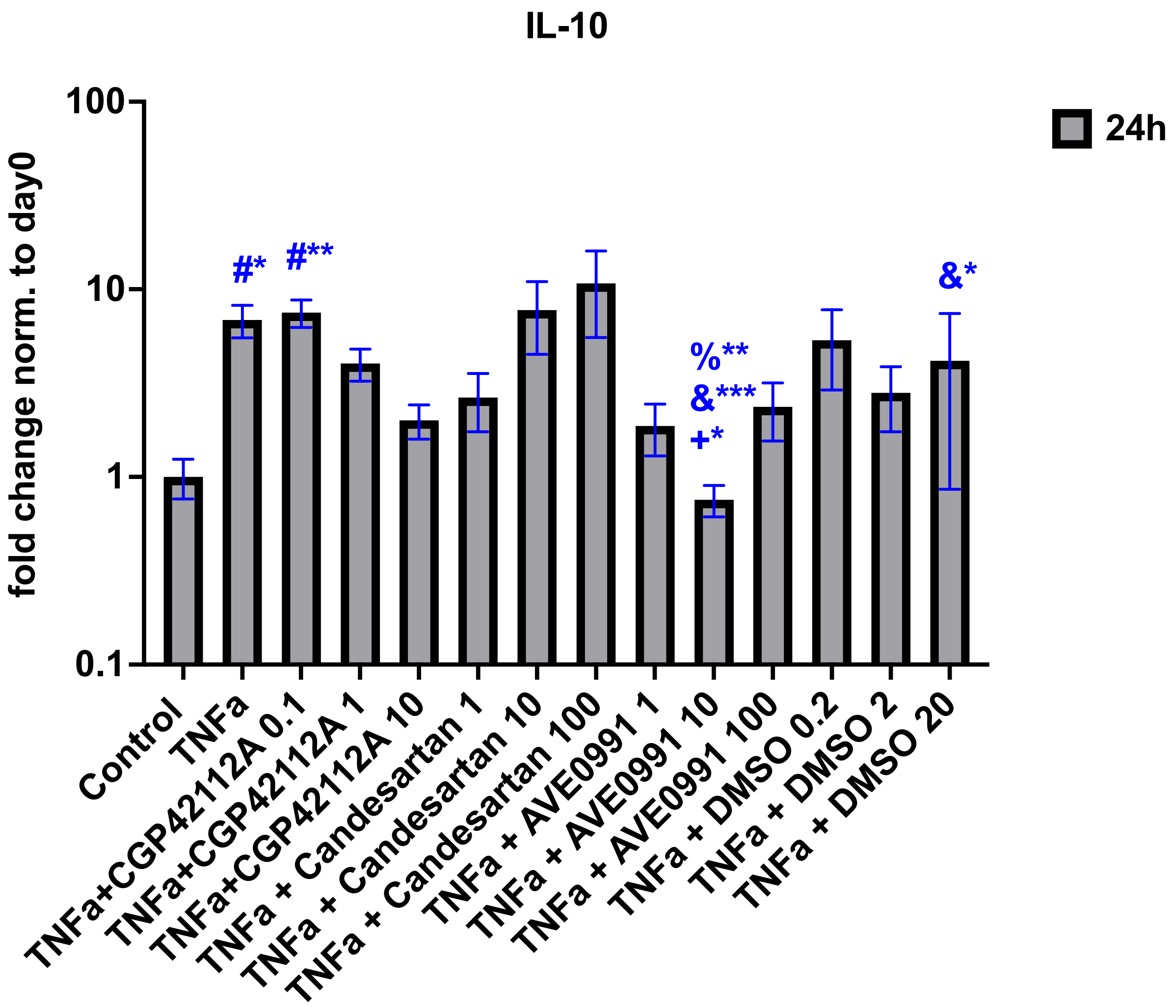

IL-10
24h
fold change norm. to day0
100
10
1
0.1
#*
#**
%**
&***
+*
&*
Control
TNFa
TNFa+CGP42112A 0.1
TNFa+CGP42112A 1
TNFa+CGP42112A 10
TNFa + Candesartan 1
TNFa + Candesartan 10
TNFa + Candesartan 100
TNFa + AVE0991 1
TNFa + AVE0991 10
TNFa + AVE0991 100
TNFa + DMSO 0.2
TNFa + DMSO 2
TNFa + DMSO 20

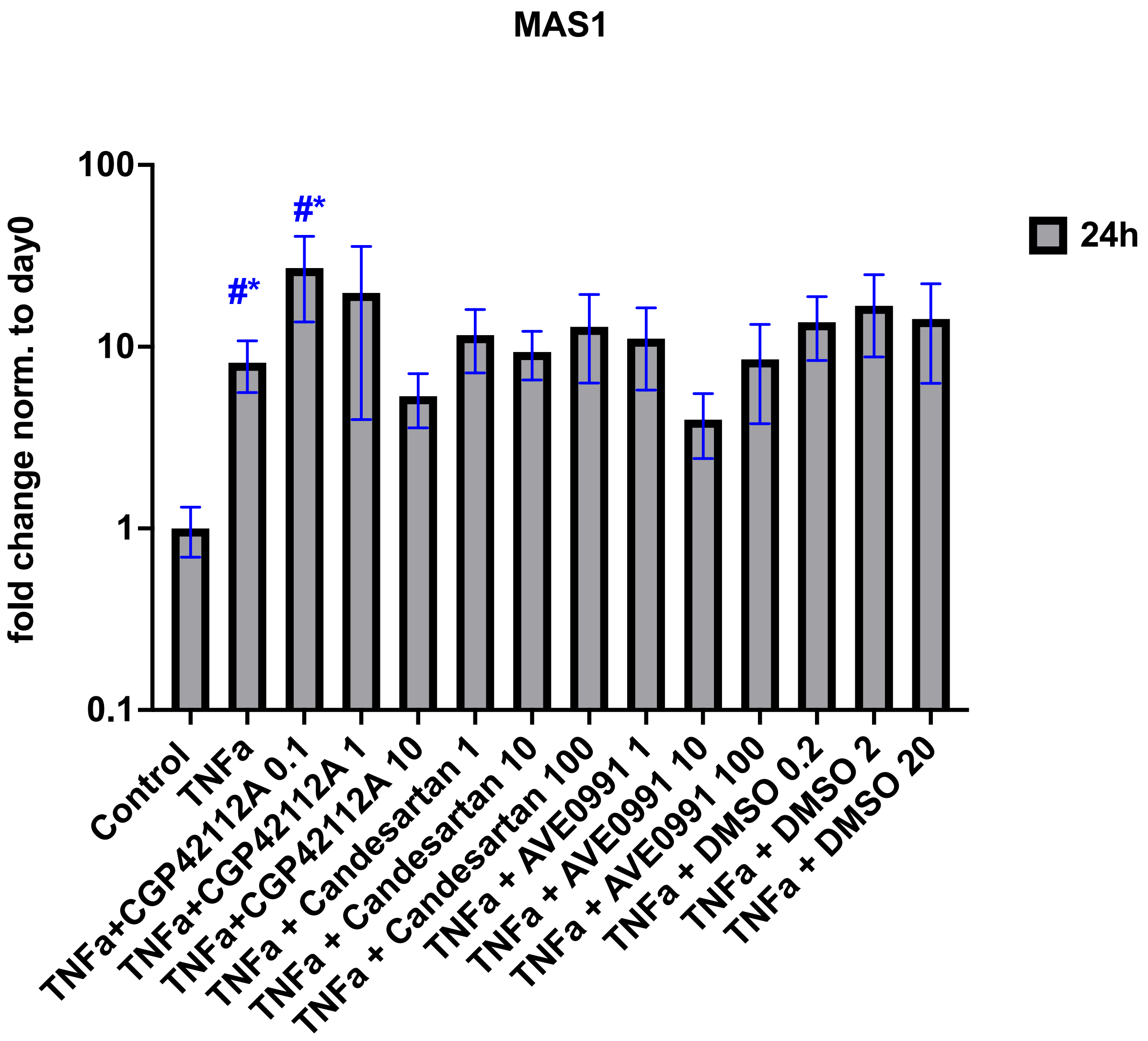
MAS1
fold change norm. to day0
100
10
1
0.1
#*
#*
24h
Control
TNFa
TNFa+CGP42112A 0.1
TNFa+CGP42112A 1
TNFa+CGP42112A 10
TNFa + Candesartan 1
TNFa + Candesartan 10
TNFa + Candesartan 100
TNFa + AVE0991 1
TNFa + AVE0991 10
TNFa + AVE0991 100
TNFa + DMSO 0.2
TNFa + DMSO 2
TNFa + DMSO 20

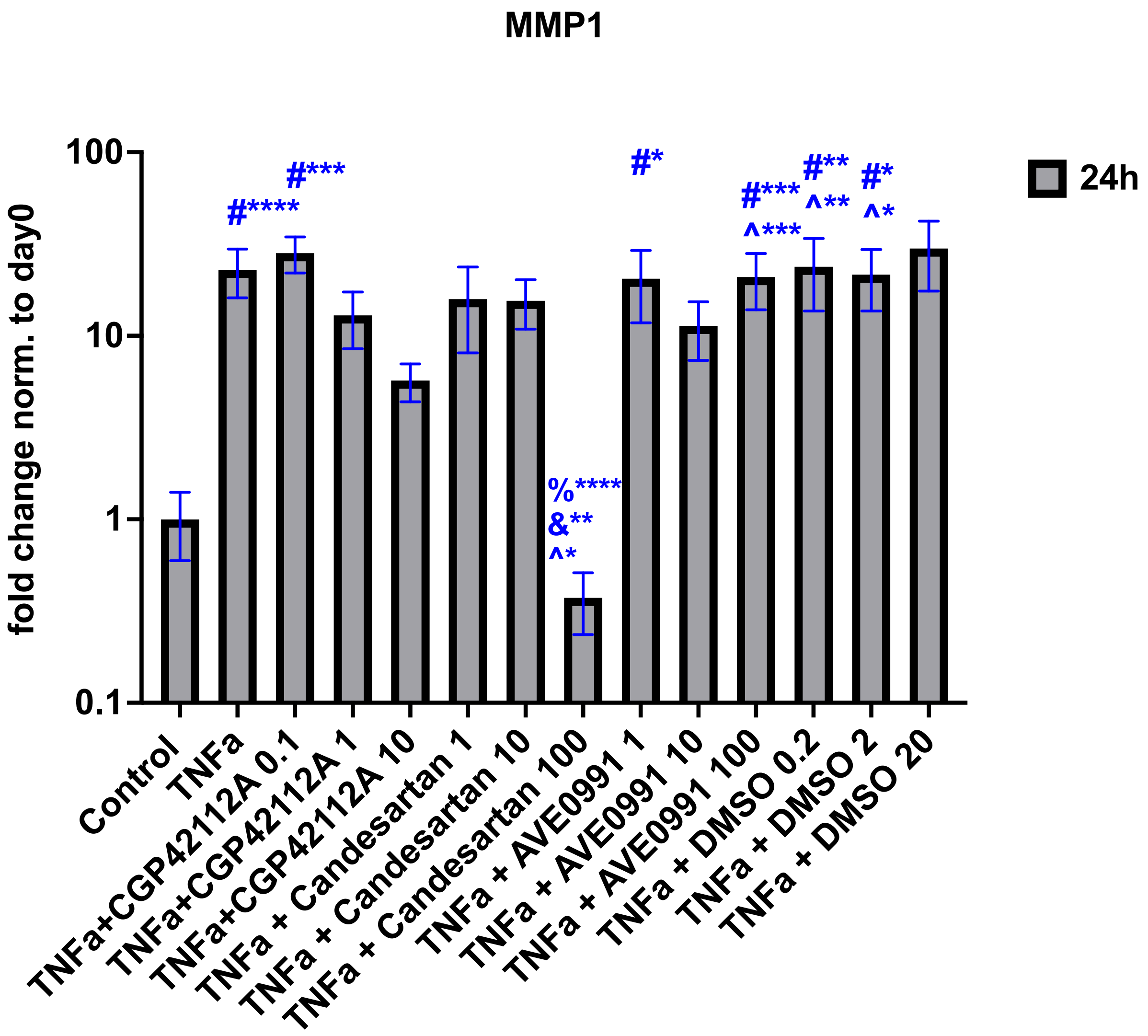

MMP1
fold change norm. to day0
100
10
1
0.1
24h
#****
#***
%****
&**
^*
#*
#***
^***
#**
^**
#*
^*
Control
TNFa
TNFa+CGP42112A 0.1
TNFa+CGP42112A 1
TNFa+CGP42112A 10
TNFa + Candesartan 1
TNFa + Candesartan 10
TNFa + Candesartan 100
TNFa + AVE0991 1
TNFa + AVE0991 10
TNFa + AVE0991 100
TNFa + DMSO 0.2
TNFa + DMSO 2
TNFa + DMSO 20

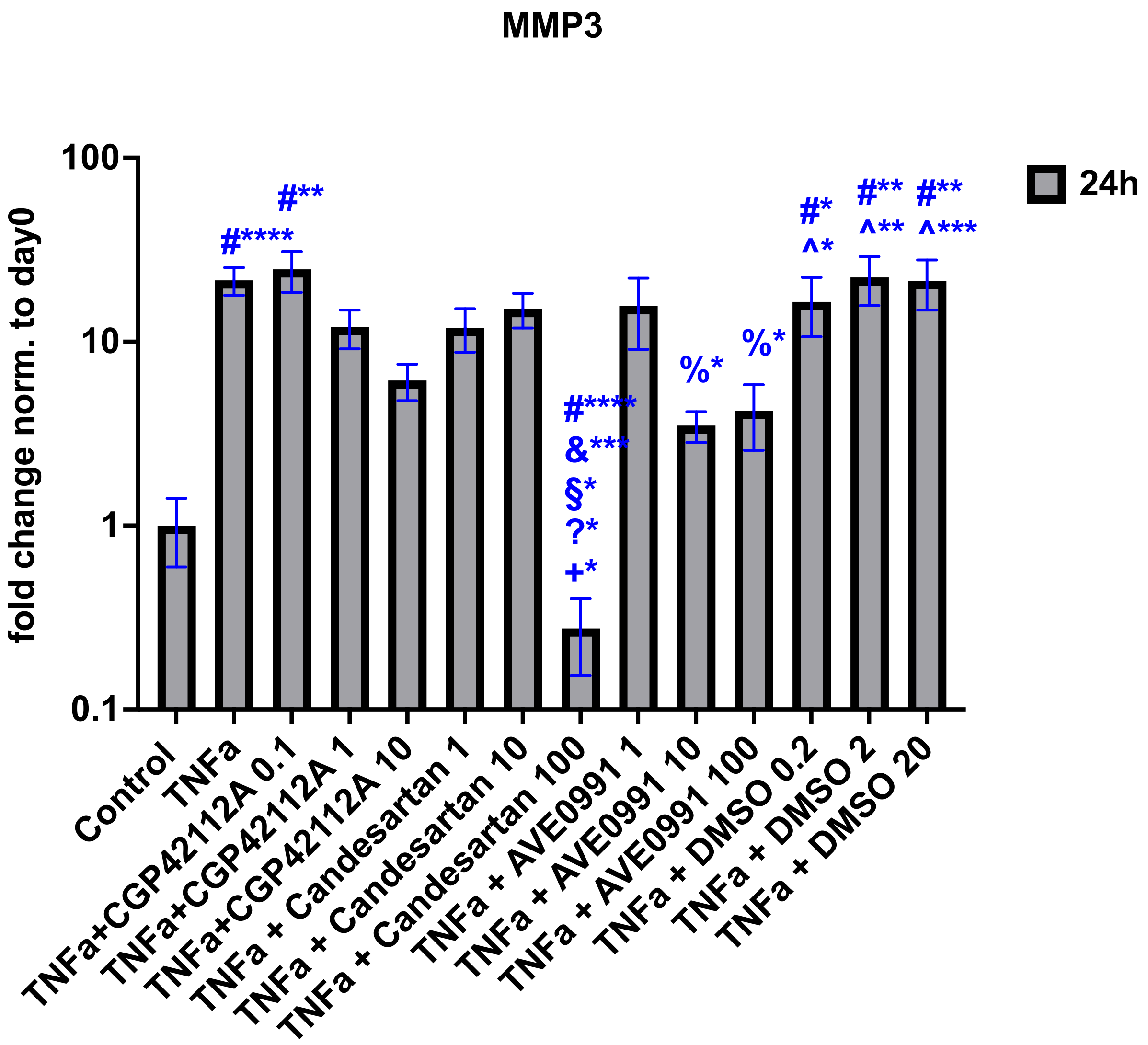
MMP3
24h
fold change norm. to day0
100
10
1
0.1
#****
#**
#****
&***
§*
?*
+*
%*
%*
#*
^*
#**
^**
#**
^***
Control
TNFa
TNFa+CGP42112A 0.1
TNFa+CGP42112A 1
TNFa+CGP42112A 10
TNFa + Candesartan 1
TNFa + Candesartan 10
TNFa + Candesartan 100
TNFa + AVE0991 1
TNFa + AVE0991 10
TNFa + AVE0991 100
TNFa + DMSO 0.2
TNFa + DMSO 2
TNFa + DMSO 20

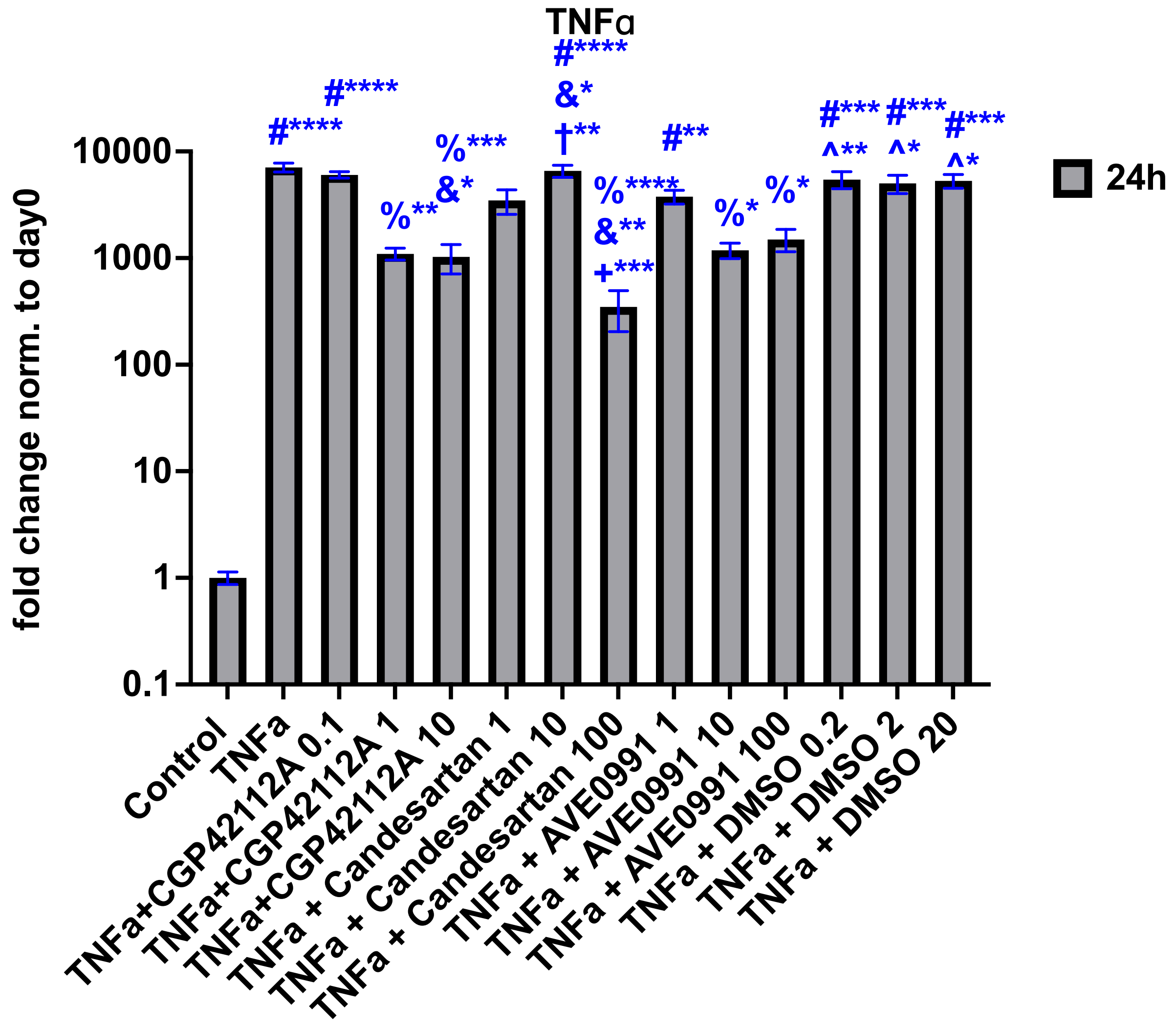

Supplementary Figure S4. Gene expression analysis in human NP cells for Candesartan (1µM, 10µM, 100µM), the MAS-receptor agonist AVE 0991 (1µM, 10µM, 100µM), and the AGTR2-receptor agonist CGP-42112A (0.1µM, 1µM, 10µM) with 24 h of exposure in in-flammatory settings (TNF-α addition). DMSO (0.1µL/ml, 1µL/ml, 10µL/ml; shown in µL volume on the x-axis) was used as the solvent control. Fold change values normalized to the baseline (day 0) are shown. n=4 biological replicates and n=3 technical replicates per donor were assessed. Mean and standard error of the mean (SEM) are shown. #: compared to Control; %: compared to TNF-α; &: compared to TNF-α+CGP42122A 0.1 µM; §: compared to TNF-α+CGP42122A 1 µM; †: compared to TNF-α+CGP42122A 10 µM; ?: compared to TNF-α+Candesartan 1 µM; +: com-pared to TNF-alpha+Candesartan 10 µM; ^: compared to TNF-α+Candesartan 100 µM; $: com-pared to TNF-alpha+AVE0991 10 µM; !: compared to TNF-alpha+AVE0991 100 µM. * $p < 0.05$; ** $p < 0.01$; *** $p < 0.001$; **** $p < 0.0001$.
